# TGF-β1/IL-11/MEK/ERK signaling mediates senescence-associated pulmonary fibrosis in a stress-induced premature senescence model of *Bmi-1* deficiency

**DOI:** 10.1038/s12276-019-0371-7

**Published:** 2020-01-21

**Authors:** Haiyun Chen, Hongjie Chen, Jialong Liang, Xin Gu, Jiawen Zhou, Chunfeng Xie, Xianhui Lv, Rong Wang, Qing Li, Zhiyuan Mao, Haijian Sun, Guoping Zuo, Dengshun Miao, Jianliang Jin

**Affiliations:** 1 Research Center for Bone and Stem Cells, Department of Human Anatomy; Key Laboratory for Aging & Disease; The State Key Laboratory of Reproductive Medicine, Nanjing Medical University, Nanjing, Jiangsu 211166 China; 2 Anti-aging Research Laboratory, Friendship Plastic Surgery Hospital, Nanjing Medical University, Nanjing, Jiangsu 210029 China; 3 Department of Nutrition and Food Safety, School of Public Health, Nanjing Medical University, Nanjing, Jiangsu 211166 China; 4 Department of Science and Technology, Jiangsu Jiankang Vocational College, Nanjing, Jiangsu 210029 China; 5 The Laboratory Center for Basic Medical Sciences, Nanjing Medical University, Nanjing, Jiangsu 211166 China

**Keywords:** Respiratory tract diseases, Experimental models of disease, Cells, Growth factor signalling, Senescence

## Abstract

To study whether TGF-β1/IL-11/MEK/ERK (TIME) signaling mediates senescence-associated pulmonary fibrosis (SAPF) in *Bmi-1-*deficient (*Bmi-1*^*−/−*^) mice and determines the major downstream mediator of Bmi-1 and crosstalk between p16^INK4a^ and reactive oxygen species that regulates SAPF, phenotypes were compared among 7-week-old *p16*^*INK4a*^ and *Bmi-1* double-knockout, *N*-acetylcysteine (NAC)-treated *Bmi-1*^*−/−*^, *Bmi-1*^*−/−*^, and wild-type mice. Pulmonary fibroblasts and alveolar type II epithelial (AT2) cells were used for experiments. Human pulmonary tissues were tested for type Ι collagen, α-smooth muscle actin (α-SMA), p16^INK4a^, p53, p21, and TIME signaling by using enzyme-linked immunosorbent assay (ELISA). Our results demonstrated that *Bmi-1* deficiency resulted in a shortened lifespan, ventilatory resistance, poor ventilatory compliance, and SAPF, including cell senescence, DNA damage, a senescence-associated secretory phenotype and collagen overdeposition that was mediated by the upregulation of TIME signaling. The signaling stimulated cell senescence, senescence-related secretion of TGF-β1 and IL-11 and production of collagen 1 by pulmonary fibroblasts and the epithelial-to-mesenchymal transition of AT2 cells. These processes were inhibited by anti-IL-11 or the MEK inhibitor PD98059. NAC treatment prolonged the lifespan and ameliorated pulmonary dysfunction and SAPF by downregulating TIME signaling more than *p16*^*INK4a*^ deletion by inhibiting oxidative stress and DNA damage and promoting ubiquitin-proteasome degradation of p16^INK4a^ and p53. Cytoplasmic p16^INK4a^ accumulation upregulated MEK/ERK signaling by inhibiting the translocation of pERK1/2 (Thr202/Tyr204) from the cytoplasm to the nucleus in senescent fibroblasts. The accumulation of collagen 1 and α-SMA in human lungs accompanied by cell senescence may be mediated by TIME signaling. Thus, this signaling in aging fibroblasts or AT2 cells could be a therapeutic target for preventing SAPF.

## Lung fibrosis: calling TIME on disease progression

Targeting cellular signals that are increased in lung fibrosis may help halt disease progression. The build-up of scarred and thickened tissues in the lungs associated with aging and cellular deterioration is known as senescence-associated pulmonary fibrosis (SAPF). There are limited treatment options, and Jianliang Jin at Nanjing Medical University, China, and co-workers believe that targeting a complex of cellular signaling pathways called TGF-β1/IL-11/MEK/ERK (TIME) signals, the disruption of which affects tissue homeostasis, may hold the key to tackling the disease. The team conducted experiments on mouse models of SAPF, deficient in a gene called *Bmi-1*. The protein encoded by *Bmi-1* is critical for cell division and DNA damage repair. In the *Bmi-1*-deficient mice, the researchers found that TIME signals were overexpressed, resulting in premature cellular deterioration, increased inflammation and accelerated collagen production.

## Introduction

Aging drives idiopathic pulmonary fibrosis (IPF) and non-IPF lung fibrotic disorders, which are characterized by chronic activation of profibrotic factors and pulmonary parenchymal destruction and dysfunction, which leads to poor health and truncation of the lifespan^1,2^. Determining the mechanism of senescence-associated pulmonary fibrosis (SAPF) is critical for developing more-effective therapies.

The senescence-associated secretory phenotype (SASP) turns senescent fibroblasts into proinflammatory cells that induces senescence and the epithelial-to-mesenchymal transition (EMT) of nearby epithelial cells^3–5^. SAPF is partly characterized by dysfunction and retention of p16^INK4a^ (hereafter termed p16)-positive and SASP-positive fibroblasts and epithelial cells in IPF. SAPF can be ameliorated by senescent cell clearance^1^. TGF-β1, a principal profibrotic factor, predisposes the aged to the development of pulmonary fibrosis, but its inhibition is associated with side effects owing to its pleiotropic roles^6,7^. IL-11 is a downstream effector of TGF-β1 and, along with its receptor α1 (Rα1), drives noncanonical MEK–ERK–RSK signals that are required for fibrogenic protein synthesis in cardiovascular fibrosis^6^. *IL-11* is upregulated 100-fold in fibroblasts from patients with IPF^8^. Senescence and the EMT of alveolar type II epithelial (AT2) cells are important in pulmonary fibrosis, leading to diminished regenerative repair of the injured epithelium owing to the EMT of AT2 cells^9^. Thus, studying whether TGF-β1/IL-11/MEK/ERK (TIME) signaling mediates SAPF by promoting the profibrotic SASP of fibroblasts and the EMT of AT2 cells is urgent.

B-cell-specific Moloney murine leukemia virus insertion region 1 (Bmi-1) is implicated in cell cycle regulation and senescence. Bmi-1 inhibits the p16/Rb and p19/p53 pathways and maintains mitochondrial function and redox balance^4,10^. *Bmi-1*-deficient mice are a stress-induced premature senescence (SIPS) model. These mice show persistent accumulation of reactive oxygen species (ROS) that results from impaired mitochondrial function and imbalanced redox and is sufficient to induce cell senescence via accumulated ROS and DNA damage^4,5,11–13^. We reported that Bmi-1 protected against renal interstitial fibrosis mediated by TGF-β1/Smad signaling by maintaining redox balance and inhibiting overproduction of p16-positive and SASP-positive fibroblasts and the EMT of tubular epithelial cells^4,5^. However, whether Bmi-1 ameliorates SAPF by inhibiting the profibrotic SASP of fibroblasts and the EMT of AT2 cells that are mediated by TIME signals is unclear. The major downstream mediator of Bmi-1 and crosstalk between p16 and ROS in regulating SAPF is also unknown.

To address these issues, *Bmi-1* and *p16* double-knockout (*Bmi-1*^*−/−*^*p16*^*−/−*^) mice were generated, and 3-week-old *Bmi-1*^*−/−*^ mice were treated with *N*-acetylcysteine (NAC). Pulmonary phenotypes were compared between *Bmi-1*^*−/−*^ and wild-type (WT) mice. Pulmonary fibroblasts and AT2 cells from the mice and samples of human pulmonary tissues were used for experiments.

**Table 1:** Primer for real-time RT-PCR.

Name	S/AS	Sequence	Species	Tm (°C)	Length (bp)
*Bmi-1*	S	5′-CTGATGCTGCCAATGGCTCC-3′	Mouse	60	198
	AS	5′-AGTCATTGCTGCTGGGCATC-3′			
*p16*	S	5′-CCCGATTCAGGTGATGATGAT-3′	Mouse	55	100
	AS	5′-GCGGGAGAAGGTAGTGG-3′			
*IL-1α*	S	5′-CGAAGACTACAGTTCTGCCATT-3′	Mouse	60	128
	AS	5′-GACGTTTCAGAGGTTCTCAGAG-3′			
*IL-1β*	S	5′-CTGGTACATCAGCACCTCAC-3′	Mouse	60	124
	AS	5′-AGAAACAGTCCAGCCCATAC-3′			
*IL-6*	S	5′-TGTATGAACAACGATGATGCACTT-3′	Mouse	60	197
	AS	5′-ACTCTGGCTTTGTCTTTCTTGTTATCT-3′			
*TNF-α*	S	5′-AGTGACAAGCCTGTAGCCC-3′	Mouse	60	252
	AS	5′-GAGGTTGACTTTCTCCTGGTAT-3′			
*Col1α1*	S	5′-CATTGTGTATGCAGCTGACTTC-3′	Mouse	59	135
	AS	5′-CGCAAAGAGTCTACATGTCTAGG-3′			
*Col1α2*	S	5′-GTAACTTCGTGCCTAGCAACA-3′	Mouse	60	234
	AS	5′-CCTTTGTCAGAATACTGAGCAGC-3′			
*ACTA2*	S	5′-GTCCCAGACATCAGGGAGTAA-3′	Mouse	60	103
	AS	5′-TCGGATACTTCAGCGTCAGGA-3′			
*PAI-1*	S	5′-TTCACAAGTCTTTCCGACCAA-3′	Mouse	56	121
	AS	5′-GGGCTGAGATGACAAAGG-3′			
*TGF-β1*	S	5′-TAAAATCAAGTGTGGAGCAAC-3′	Mouse	55	119
	AS	5′-GTCAAAAGACAGCCACTCAG-3′			
*MMP9*	S	5′-TCTACAGAGTCTTTGAGTCCG-3′	Mouse	55	134
	AS	5′-GGGCTTCCTCTATGATTCAG-3′			
*MMP12*	S	5′-GCTCCTGCCTCACATCATAC-3′	Mouse	59	112
	AS	5′-GGCTTCTCTGCATCTGTGAA-3′			
*IL-11*	S	5′-TGTTCTCCTAACCCGATCCCT-3′	Mouse	60	149
	AS	5′-CAGGAAGCTGCAAAGATCCCA-3′			
*IL-11Rα1*	S	5′-CTCTTGCCAAGCGGTAGACTA-3′	Mouse	60	139
	AS	5′-GGATGGACTTTCCCTCTGACTC-3′			
*SFTPC*	S	5′-ATGGACATGAGTAGCAAAGAGGT-3′	Mouse	60	117
	AS	5′-CACGATGAGAAGGCGTTTGAG-3′			
*β-Actin*	S	5′-GGCTGTATTCCCCTCCATCG-3′	Mouse	60	154
	AS	5′-CCAGTTGGTAACAATGCCATGT-3′			

*S* sense, *AS* antisense sequence, *Tm* annealing temperature, *Length* amplicon

**Table 2:** Lifetime survival studies.

Age-interval (Day)	Number entering the age interval (Nx)				Number dead within the age interval (Dx)				Number censored within the age interval (Cx)			
	WT	KO	DKO	KO + NAC	WT	KO	DKO	KO + NAC	WT	KO	DKO	KO + NAC
0–7	25	46	22	8	0	0	0	0	25	46	22	8
8–14	25	46	22	8	0	4	0	0	25	46	22	8
15–21	25	42	22	8	0	8	0	0	25	42	22	8
22–28	25	34	22	8	0	7	0	0	25	34	22	8
29–35	25	27	22	8	0	12	0	0	25	27	22	8
36–42	25	15	22	8	0	4	1	0	25	15	22	8
43–49	25	11	21	8	0	3	1	0	25	11	21	8
50–56	25	8	20	8	0	3	3	0	25	8	20	8
57–63	25	5	17	8	0	3	2	0	25	5	17	8
64–70	25	2	15	8	0	1	2	1	25	2	15	8
71–77	25	1	13	7	0	0	0	0	25	1	13	7
78–84	25	1	13	7	0	1	1	0	25	1	13	7
85–91	25	0	12	7	0	0	2	0	25	0	12	7
92–98	25	0	10	7	0	0	2	1	25	0	10	7
99–105	25	0	8	6	0	0	0	0	25	0	8	6
106–112	25	0	8	6	0	0	1	0	25	0	8	6
113–119	25	0	7	6	0	0	1	2	25	0	7	6
120–126	25	0	6	4	0	0	0	0	25	0	6	4
127–133	25	0	6	4	0	0	2	0	25	0	6	4
134–140	25	0	4	4	0	0	1	0	25	0	4	4
141–147	25	0	3	4	0	0	0	1	25	0	3	4
148–154	25	0	3	3	0	0	0	0	25	0	3	3
155–161	25	0	3	3	0	0	0	1	25	0	3	3
162–168	25	0	3	2	0	0	2	1	25	0	3	2
169–175	25	0	1	1	0	0	1	0	25	0	1	1
176–182	25	0	0	1	0	0	0	1	25	0	0	1
183–189	25	0	0	0	0	0	0	0	25	0	0	0

## Materials and methods

### Mice and genotyping

*Bmi-1*^*−/−*^*p16*^*−/−*^, *Bmi-1*^*−/−*^, and WT mice were prepared as described previously^4,5^. This study was carried out in strict accordance with the guidelines of the Institute for Laboratory Animal Research of Nanjing Medical University in Nanjing, China. The protocol was approved by the Committee on the Ethics of Animal Experiments of Nanjing Medical University (Permit Number: IACUC-1706001).

### Samples of human pulmonary tissues

Human pulmonary samples were obtained from 27 autopsies at the Department of Human Anatomy in Nanjing Medical University. Anatomical methods and all experimental protocols were approved by the Committee on the Ethics of Nanjing Medical University (Permit Number: 2019-902). Body donors, aged 39–94 years old, had no tumors, acquired immune deficiency syndrome, autoimmune disease, chronic respiratory infections or inflammatory diseases before they died.

### Cell cultures

#### Pulmonary fibroblasts

Mice that were 7 weeks old were anesthetized and perfused. The lungs were separated, minced, and digested to culture pulmonary fibroblasts that were detected by immunofluorescence staining of the mesenchymal cell marker vimentin (Fig. 4e) as described previously^5,14^. Details are provided in Supporting Information 3.

#### AT2 cells

AT2 cells were separated, cultured and detected by western blotting and immunofluorescence for the marker surfactant protein C (SFTPC) (Fig. 6d, f, h, j) as described previously^15–17^. Details are provided in Supporting Information 3.

### Administration of drugs or reagents

#### *N*-acetylcysteine

In vivo, NAC was administered at 1 mg/ml in drinking water as previously described^4,12^.

In vitro, NAC was used at 1 mm (0.163 mg/ml) as previously described^4,18^. Pulmonary fibroblasts or AT2 cells in the NAC-treated *Bmi-1*^−/−^ group were isolated from NAC-treated *Bmi-1*^−/−^ mice and continuously cultured with NAC.

#### Exogenous recombinant mouse TGF-β1 and IL-11, the MEK inhibitor and anti-IL-11 antibodies

Cells were treated with TGF-β1 (5 ng/ml, 48 h) (Novoprotein Scientific Inc., Shanghai, China), anti-IL-11 (2 μg/ml, 48 h) (sc-133063, Santa Cruz Biotechnology Inc., Dallas, TX, USA), IL-11 (5 ng/ml, 48 h) (Novoprotein Scientific Inc.), or a MEK inhibitor (PD98059) (10 μm, 48 h) (#9900, Cell Signaling Technology, Beverly, MA, USA) as previously described^6^.

#### MG132

MG132 (#474787, Sigma-Aldrich, St. Louis, MO, USA) was used at 5 μm for 48 h as previously described^19^.

### Cell proliferation

Cell proliferation was analyzed using a Cell Counting Kit-8 assay (#C0038, Beyotime Institute of Biotechnology, Shanghai, China) as previously described^5^. Details are provided in Supporting Information 3.

### CM collection

Third-passage pulmonary fibroblasts were cultured in Dulbecco's Modified Eagle Medium/F12 (without phenol red; Gibco, Life Technologies Corporation, NY, USA) without FBS for 24 h. CM was collected as previously described^5^. Details are provided in Supporting Information 3.

### Enzyme-linked immunosorbent assay

ELISA kits (Yifeixue Biotechnology, Nanjing, China) were used to detect concentrations of mouse-derived TGF-β1 (#M00081) and IL-11 (#M00807) in the CM and serum of mice and human-derived TGF-β1 (#H00080), IL-11 (#H00439), p16 (#H01273), collagen 1 (#H00179), pERK1/2 (Thr202/Tyr204) (#H01101), p53 (#H00199), p21 (#H00200) and α-smooth muscle actin (α-SMA) (#H01041) in human pulmonary tissue.

### Intracellular ROS analysis

Intracellular ROS analysis was performed as previously described^4,12^. Details are provided in Supporting Information 3.

### Pulmonary function analysis

Mice that were 7 weeks old were anesthetized and underwent tracheostomies. They were mechanically ventilated at an initial baseline challenge using the FinePointe RC system (Buxco Research Systems, Wilmington, NC, USA) to directly evaluate lung ventilatory resistance and compliance, including peak inspiratory flow, frequency, tidal volume, lung resistance, dynamic compliance, minute volume, static compliance, and elastance^20–22^.

### Preparation of pulmonary sections

Mice that were 7 weeks old were anesthetized and perfused as previously described^4^. Pulmonary samples were cut into small pieces and postfixed in periodate-lysine-paraformaldehyde (PLP) solution overnight at 4 °C as previously described^23^. For histochemistry or immunohistochemistry, sections were dehydrated in a series of graded ethanol solutions, embedded in paraffin and cut into 5-μm sections using a rotary microtome (Leica Biosystems Nussloch GmbH, Nussloch, Germany) as previously described^5^.

Human frozen pulmonary samples were cut on a freezing microtome (Thermo Scientific Cryotome FSE Cryostats, Loughborough, Leicestershire) at a thickness of 7 μm for SA-β-gal staining and α-SMA immunohistochemical staining.

### Histology staining

For histochemical or immunohistochemical staining, serial paraffin sections were deparaffinized and rehydrated.

#### Pre-embedding SA-β-gal staining

Pulmonary samples of mice were stained following previously described methods^4,5,24^.

#### SA-β-gal staining

Frozen sections of human pulmonary samples were stained by using a senescence β-Galactosidase staining kit (#C0602, Beyotime Institute of Biotechnology, Shanghai, China) according to the manufacturer’s instructions and following previously described methods^25^.

#### Masson’s trichrome staining

Serial paraffin sections were generated with Masson’s detection kits (#D026, Nanjing Jiancheng Bioengineering Institute, Nanjing, Jiangsu, China) according to the manufacturer’s instructions as previously described^4,5^.

#### Immunohistochemical staining

Staining was performed as previously described^4,5,23^. Primary antibodies against p53 (#2524, Cell Signaling Technology, Beverly, MA, USA), 8-OHdG (ab62623, Abcam, Cambridge, MA, USA), IL-1β (ab9722, Abcam), IL-6 (sc-1265, Santa Cruz Biotechnology Inc., Dallas, TX, USA), TNF-α (sc-52746, Santa Cruz Biotechnology Inc.), α-SMA (ab28052, Abcam), collagen 1 (#1310-08, Southern Biotech, Birmingham, AL, USA), fibronectin (#SAB4500974, Sigma-Aldrich), TGF-β1 (ab64715, Abcam), and IL-11 (sc-133063, Santa Cruz Biotechnology Inc.). After washing, the sections were incubated with secondary antibody (biotinylated IgG; Sigma-Aldrich), washed and processed using Vectastain ABC-HRP kits (Vector Laboratories Inc., Burlingame, CA, USA).

#### Immunofluorescent staining of pulmonary sections

Primary antibodies against CD3 (sc-20047, Santa Cruz Biotechnology Inc.), F4/80 (sc-377009, Santa Cruz Biotechnology Inc.), IL-11Rα1 (sc-130920, Santa Cruz Biotechnology Inc.), SFTPC (ab211326, Abcam), TGF-β1 (ab64715, Abcam), IL-11 (sc-133063, Santa Cruz Biotechnology Inc.), vimentin (#5741, Cell Signaling Technology), p53 (#2524, Cell Signaling Technology), p16 (#MA5-17142, Invitrogen Inc.), fibroblast marker (ER-TR7) (sc-73355, Santa Cruz Biotechnology Inc.), and TGF-β RII (sc-17792, Santa Cruz Biotechnology Inc.) and affinity-purified Alexa Fluor 488-conjugated and 594-conjugated secondary antibodies (Life Technologies Corporation, USA) were used. Details are provided in Supporting Information 3.

### Cytology staining

Cells seeded on a Lab-Tek II Chamber Slide system (Thermo Fisher Scientific Inc., Rochester, NY, USA) were fixed with PLP solution for 1 h^5,26^.

#### SA-β-gal staining

SA-β-gal staining of cells was performed by using the senescence β-Galactosidase staining kit (#C0602, Beyotime Institute of Biotechnology) according to the manufacturer’s instructions as previously described^25^.

#### Immunofluorescent staining of cells

Primary antibodies against IL-11 (sc-133063, Santa Cruz Biotechnology Inc.), vimentin (#5741, Cell Signaling Technology), p53 (#2524, Cell Signaling Technology), TGF-β RII (sc-17792, Santa Cruz Biotechnology Inc.), IL-11Rα1 (sc-130920, Santa Cruz Biotechnology Inc.), α-SMA (ab28052, Abcam), SFTPC (ab211326, Abcam), p16 (#MA5-17142, Invitrogen Inc.), ERK1/2 (#4695, Cell Signaling Technology), and pERK1/2(Thr202/Tyr204) (#4370, Cell Signaling Technology) and affinity-purified Alexa Fluor 488-conjugated and 594-conjugated secondary antibodies (Life Technologies Corporation, USA) were used. Details are provided in Supporting Information 3.

### RNA extraction and real-time RT-PCR

RNA was extracted from the lungs of 7-week-old mice using TRIzol reagent (#15596, Invitrogen Inc.) according to the manufacturer’s protocol. Levels of mRNA in pulmonary samples were quantified by real-time RT-PCR as previously described^4,5^. Primers are listed in Table 1.

### Western blots

Western blots were generated as previously described^5,26^. Primary antibodies against p16 (ab211542, Abcam), p19 (sc-1665, Santa Cruz Biotechnology Inc.), p53 (sc-126, Santa Cruz Biotechnology Inc.), p21 (sc-471, Santa Cruz Biotechnology Inc.), 8-OHdG (ab62623, Abcam), SFTPC (ab211326, Abcam), collagen 1 (#1310-08, Southern Biotech), α-SMA (ab28052, Abcam), TGF-β1 (ab64715, Abcam), TGF-β RII (sc-17792, Santa Cruz Biotechnology Inc.), Smad2 (sc-101153, Santa Cruz Biotechnology Inc.), phospho-Smad2 (Ser465/467) (#3108, Cell Signaling Technology), phospho-Smad2/3 (Ser423/425) (sc-11769, Santa Cruz Biotechnology Inc.), IL-11 (sc-133063, Santa Cruz Biotechnology Inc.), IL-11Rα1 (sc-130920, Santa Cruz Biotechnology Inc.), MEK1/2 (sc-81504, Santa Cruz Biotechnology Inc.), phospho-MEK1/2 (sc-81503, Santa Cruz Biotechnology Inc.), ERK1/2 (#4695, Cell Signaling Technology), pERK1/2 (Thr202/Tyr204) (#4370, Cell Signaling Technology), eIF4E (sc-9976, Santa Cruz Biotechnology Inc.), p-eIF4E (#9741, Cell Signaling Technology), RSK (sc-74575, Santa Cruz Biotechnology Inc.), p-RSK (Ser380) (sc-377526, Santa Cruz Biotechnology Inc.), or Snail (#3879, Cell Signaling Technology) were used. Histone H3 (#4499, Cell Signaling Technology) was the loading control for the nuclear fraction and β-actin (BS6007M, Bioworld Technology, St. Louis Park, MN, USA) or GAPDH (AP0063, Bioworld Technology) was the loading control for the cytoplasmic fraction and total cell protein.

### Coimmunoprecipitation

This analysis was performed with Pierce CoImmunoprecipitation (Co-IP) Kits (#26149, Pierce Biotechnology, Rockford, IL, USA) as previously described^27^. Pulmonary fibroblasts from WT mice were treated with TGF-β1 and extracted for p16 (ab211542, Abcam), Bmi-1 (#6964, Cell Signaling Technology), ERK1/2 (#4695, Cell Signaling Technology) or pERK1/2 (Thr202/Tyr204) (#4370, Cell Signaling Technology) coimmunoprecipitation analysis. Coprecipitates or total cytoplasmic lysates were detected with ERK1/2 (#4695, Cell Signaling Technology) and pERK1/2 (Thr202/Tyr204) (#4370, Cell Signaling Technology) or p16 (ab211542, Abcam) antibodies for western blots analysis.

### Duolink proximity ligation assay (PLA)

Duolink PLA in situ fluorescence (Sigma-Aldrich) was performed according to the manufacturer’s instructions with Duolink in situ PLA probe anti-mouse PLUS (#DUO92001), Duolink in situ PLA probe anti-rabbit MINUS (#DUO92005), Duolink in situ detection reagents Red (#DUO92008) and Duolink in situ wash buffers-fluorescence (#DUO82049). Pulmonary fibroblasts from *Bmi-1*^*−/−*^ mice were treated with TGF-β1 and detected with antibodies against p16 (MA5-17142, Thermo Fisher Scientific, IL, USA) and ERK1/2 (#4695, Cell Signaling Technology), and p16 (MA5-17142, Thermo Fisher Scientific, IL, USA) and pERK1/2(Thr202/Tyr204) (#4370, Cell Signaling Technology). The PLA signal (λex 594 nm, λem 624 nm; Texas Red) was analyzed as previously described^28^.

### Statistical analysis

All analyses were performed using GraphPad Prism software (Version 6.07; GraphPad Software Inc., San Diego, CA, USA) as previously described^6^. Measurement data are described as the mean ± SEM fold-change over the vehicle group and were analyzed by using Student’s *t* test and one-way ANOVA to compare differences among groups. Qualitative data are described as percentages and were analyzed using chi-square tests as indicated. *P* values were two-sided and a *P* value < 0.05 was considered statistically significant^4,5^. The correlation of Gaussian distributed data was analyzed by Pearson’s *r*, and non-Gaussian distributed data were analyzed by Spearman’s *r*. *P* values were two-sided, and a *P* value < 0.05 was considered statistically significant.

## Results

### Amelioration of premature senescence and pulmonary dysfunction by NAC in *Bmi-1*^*−/−*^ vs. *Bmi-1*^*−/−*^*p16*^*−/−*^ mice

**Fig. 1:**
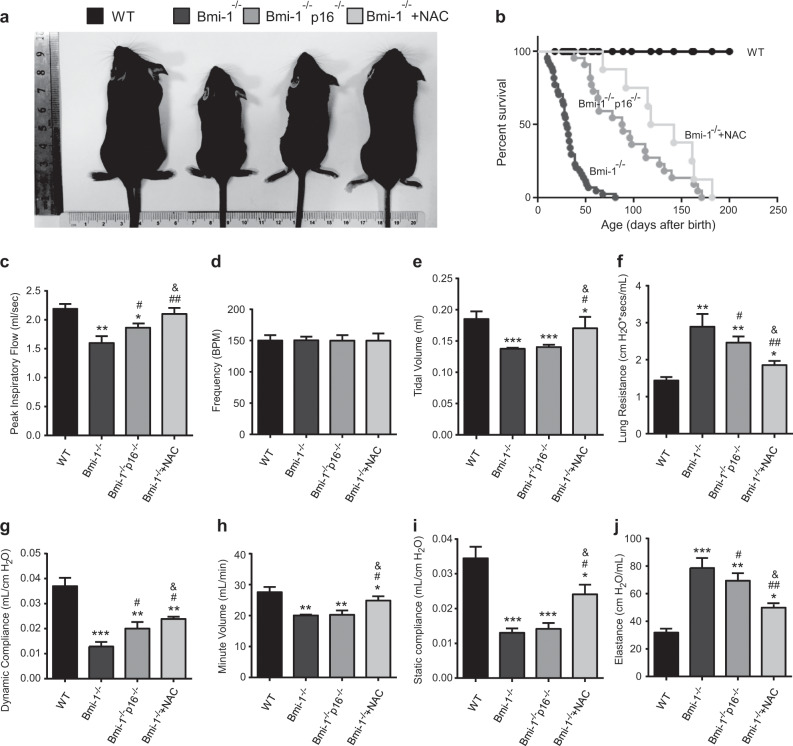
NAC treatment improved premature senescence and pulmonary dysfunction in *Bmi-1*^*−/−*^ mice better than in *Bmi-1*^*−/−*^*p16*^*−/−*^ mice. **a** Representative appearances of 7-week-old WT, *Bmi-1*^*−/−*^, *Bmi-1*^−/−^*p16*^−/−^, and NAC-treated *Bmi-1*^*−/−*^ (*Bmi-1*^*−/−*^ + NAC) mice. **b** Percent survival of the mice. Pulmonary function was detected by the Buxco measurement system and FinePointe analysis for **c** peak inspiratory flow, **d** frequency, **e** tidal volume, **f** lung resistance, **g** dynamic compliance, **h** minute volume, **i** static compliance, and **j** elastance. Six mice per group were used for experiments. Values are the means ± SEM of six determinations per group. *P < 0.05, **P < 0.01, ***P < 0.001 compared with the WT group; ^#^*P* < 0.05, ^##^*P* < 0.01 compared with the *Bmi-1*^*−/−*^ group; ^&^*P* < 0.05 compared with the *Bmi-1*^−/−^*p16*^−/−^ group.

*Bmi-1*^*−/−*^ mice had significantly decreased body sizes and survival rates (Fig. 1a, b) (Table 2). To assess pulmonary function, ventilatory resistance, and compliance indexes were determined. Peak inspiratory flow, tidal volume, dynamic compliance, minute volume, and static compliance decreased significantly, whereas lung resistance, elastance, and pulmonary ROS levels increased significantly in *Bmi-1*^*−/−*^ mice compared with those of WT mice. Pulmonary frequency was not altered in *Bmi-1*^*−/−*^ mice (Fig. 1c–j and S1). NAC treatment increased body sizes and survival rates, peak inspiratory flow, tidal volume, dynamic compliance, minute volume, and static compliance and decreased lung resistance and elastance in *Bmi-1*^*−/−*^ mice more than in *Bmi-1*^*−/−*^*p16*^*−/−*^ mice (Table 2). NAC treatment decreased pulmonary ROS levels in *Bmi-1*^*−/−*^ mice (Fig. 1c–j and S1).

### Amelioration of pulmonary cell senescence, DNA damage, and SASP by NAC in *Bmi-1*^*−/−*^ vs. *Bmi-1*^*−/−*^*p16*^*−/−*^ mice

**Fig. 2:**
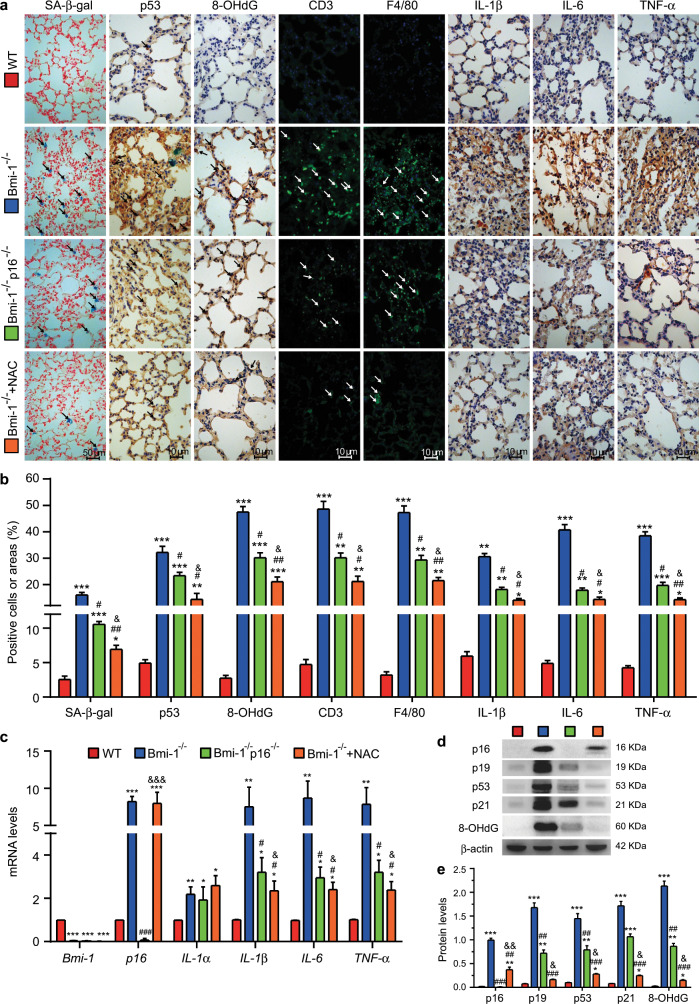
NAC treatment improved pulmonary cell senescence, DNA damage, and SASP in *Bmi-1*^*−/−*^ mice more than in *Bmi-1*^*−/−*^*p16*^*−/−*^ mice. **a** Representative micrographs of paraffin-embedded pulmonary sections stained for senescence-associated β-galactosidase (SA-β-gal), stained immunohistochemically for p53, 8-hydroxyguanosine (8-OHdG), IL-1β, IL-6, and TNF-α, and stained immunofluorescently for CD3 and F4/80. SA-β-gal-, p53-, 8-OHdG-, CD3-, or F4/80-positive cells are indicated by arrows. **b** Percentage of cells positive for SA-β-gal, p53, 8-OHdG, CD3, F4/80, IL-1β, IL-6, and TNF-α or with positive area relative to the total cells or area. **c** *Bmi-1*, *p16*, *IL-1a*, *IL-1β*, *IL-6*, and *TNF-α* mRNA levels in lungs by real-time RT-PCR, calculated as the ratio to β-actin mRNA and expressed relative to that of WT. **d** Western blots of pulmonary extracts showing p16, p19, p53, p21, and 8-OHdG. β-actin was used as the loading control. **e** Protein levels relative to β-actin were assessed by densitometric analysis. Six mice per group were used for experiments. Values are the mean ± SEM from six determinations per group. *P < 0.05, **P < 0.01, ***P < 0.001 compared with the WT group; ^#^*P* < 0.05, ^##^*P* < 0.01, ^###^*P* < 0.001 compared with the *Bmi-1*^*−/−*^ group; ^&^*P* < 0.05, ^&&^*P* < 0.01, ^&&&^*P* < 0.001 compared with the *Bmi-1*^−/−^*p16*^−/−^ group.

To determine whether the rescue of pulmonary dysfunction by NAC treatment and *p16* deletion was associated with alterations in cell senescence and associated proinflammatory secretory phenotypes, the lungs were examined for markers of senescence, DNA damage, inflammatory cell infiltration, and proinflammatory factors. The percentages of pulmonary senescence-associated β-galactosidase (SA-β-gal)-positive areas, p53-positive cells, 8-hydroxydeoxyguanosine (8-OHdG)-positive cells, CD3- and F4/80-positive inflammatory cells, and IL-1β-, IL-6-, and TNF-α-positive areas were increased significantly in *Bmi-1*^*−/−*^ mice compared with those of WT mice; mRNA expression of *p16*, *IL-1α*, *IL-1β*, *IL-6*, and *TNF-α* and protein expression of p16, p19, p53, p21, and 8-OHdG were also increased significantly in *Bmi-1*^*−/−*^ mice compared with those of WT mice. NAC treatment decreased the percentages of pulmonary SA-β-gal-positive areas, p53-positive cells, 8-OHdG-positive cells CD3- and F4/80-positive inflammatory cells and IL-1β, IL-6, and TNF-α-positive areas; NAC treatment also decreased mRNA expression of *IL-1β*, *IL-6*, and *TNF-α* and protein expression of p19, p53, p21, and 8-OHdG, and these decreases in percentages and expression were greater than those of *p16* deletion. NAC treatment also decreased p16 protein expression. These results suggest that NAC treatment had more antisenescence effects by further inhibiting the expression of p19, p53, p21, and 8-OHdG in the lungs (Fig. 2).

### Amelioration of SAPF by NAC in *Bmi-1*^*−/−*^ vs. *Bmi-1*^*−/−*^*p16*^*−/−*^ mice

**Fig. 3:**
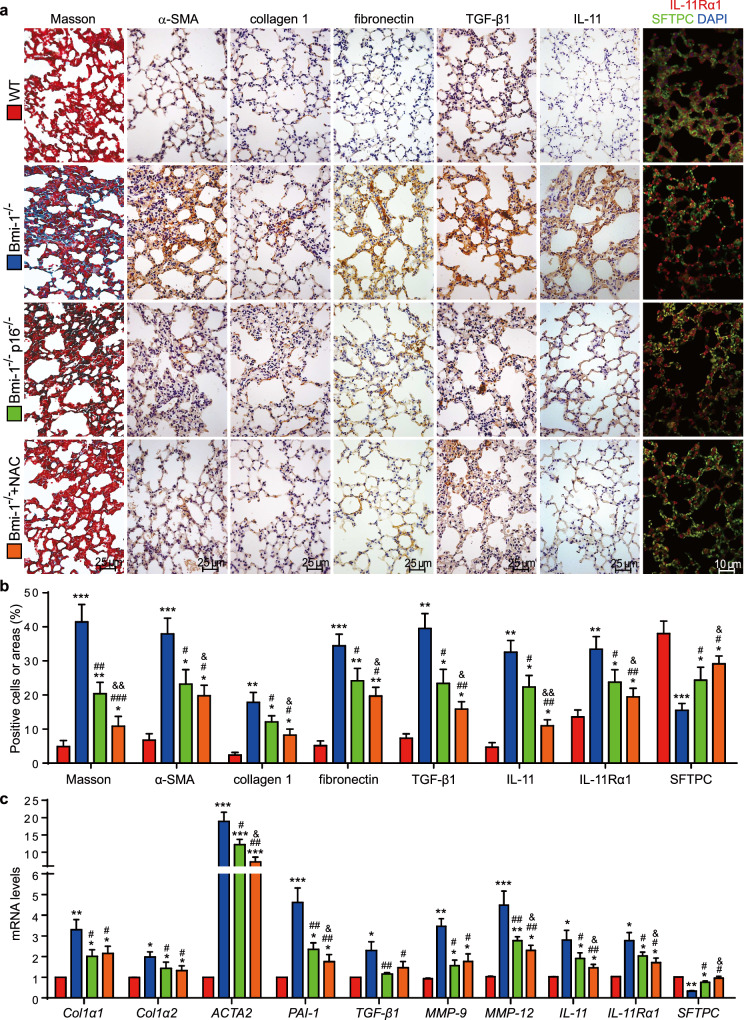
NAC treatment improved pulmonary fibrosis in *Bmi-1*^*−/−*^ mice more than in *Bmi-1*^*−/−*^*p16*^*−/−*^ mice. **a** Representative micrographs of paraffin-embedded pulmonary sections stained histochemically for Masson’s trichrome (Masson), immunohistochemically for α-smooth muscle actin (α-SMA), type Ι collagen (collagen 1), fibronectin, TGF-β1 and IL-11, and immunofluorescently for IL-11 receptor α1 (Rα1) and SFTPC, with DAPI staining the nuclei. **b** Percentage of cells positive for Masson, α-SMA, collagen 1, fibronectin, TGF-β1, IL-11, IL-11Rα1, or SFTPC or positive areas relative to the total cells or areas. **c** *Col1a1*, *Col1a2*, *ACTA2*, *plasminogen activator inhibitor-1* (*PAI-1*), *TGF-β1*, *matrix metalloproteinases* (*MMP9*), *MMP12*, *IL-11* and *IL-11Rα1* and *SFTPC* mRNA levels in lungs by real-time RT-PCR, calculated as the ratio to β-actin mRNA and expressed relative to that of WT. Six mice per group were used for experiments. Values are the mean ± SEM from six determinations per group. *P < 0.05, **P < 0.01, ***P < 0.001 compared with the WT group; ^#^*P* < 0.05, ^##^*P* < 0.01, ^###^*P* < 0.001 compared with the *Bmi-1*^*−/−*^ group; ^&^*P* < 0.05, ^&&^*P* < 0.01 compared with the *Bmi-1*^−/−^*p16*^−/−^ group.

**Fig. 4:**
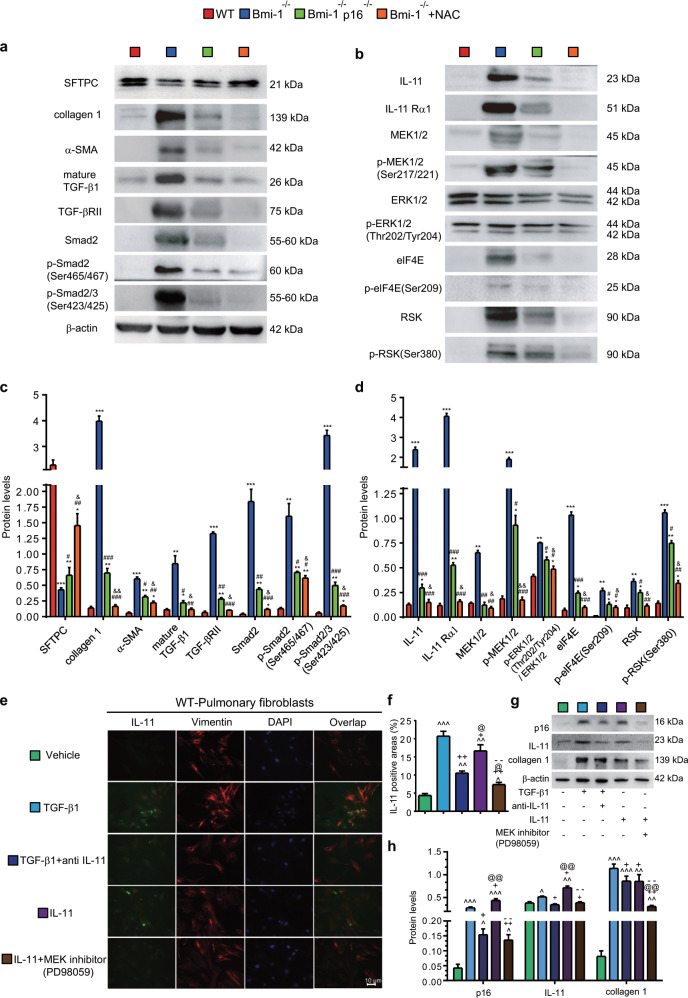
NAC treatment inhibited TIME signaling in *Bmi-1*^*−/−*^ mice more than in *Bmi-1*^*−/−*^*p16*^*−/−*^ mice. Western blots of pulmonary extracts showing **a** SFTPC, collagen 1, α-SMA, mature TGF-β1, TGF-βRII, Smad2, pSmad2 (Ser465/467), pSmad2/3 (Ser423/425); **b** IL-11, IL-11Rα1, MEK1/2, pMEK1/2(Ser217/221), ERK1/2, pERK1/2(Thr202/Tyr204), elF4E, p-elF4E(Ser209), RSK, and p-RSK(Ser380). **c**, **d** Protein expression relative to β-actin (graph a) was assessed by densitometric analysis. Six mice per group were used for experiments. Values are the means ± SEM of six determinations. *P < 0.05, **P < 0.01, ***P < 0.001 compared with the WT group; ^#^*P* < 0.05, ^##^*P* < 0.01, ^###^*P* < 0.001 compared with the *Bmi-1*^*−/−*^ group; ^&^*P* < 0.05, ^&&^*P* < 0.01 compared with the *Bmi-1*^−/−^*p16*^−/−^ group. **e** Pulmonary fibroblasts from 7-week-old WT mice treated with TGF-β1 (5 ng/ml, 48 h), TGF-β1 (5 ng/ml, 48 h) and anti-IL-11 antibody (2 μg/ml, 48 h), IL-11 (5 ng/ml, 48 h), or IL-11 (5 ng/ml, 48 h) and the MEK inhibitor (PD98059) (10 μm, 48 h). Representative micrographs immunofluorescently labeled for IL-11 and vimentin, with DAPI staining the nuclei. **f** Percentage of IL-11-positive areas relative to the total. **g** Western blots showing p16, IL-11, and collagen 1. β-actin was used as the loading control. **h** Protein expression relative to β-actin was assessed by densitometric analysis. Six biological replicates were used per experiment. Values are the means ± SEM of six determinations. ^^^*P* < 0.05, ^^^^*P* < 0.01, ^^^^^*P* < 0.001 compared with the vehicle group; ^+^*P* < 0.05, ^++^*P* < 0.01 compared with the TGF-β1 group; ^@^*P* < 0.05, ^@@^*P* < 0.01 compared with the TGF-β1 and anti-IL-11 group; ^−−^*P* < 0.01 compared with the IL-11 group.

To investigate whether pulmonary fibrosis is caused by cell senescence, DNA damage and SASP and ameliorated by NAC or *p16* deletion, the lungs were examined for fibrosis markers and AT2 cells. In *Bmi-1*^*−/−*^ mice, significant decreases were observed in Masson’s trichrome (Masson)-labeled interstitial fibers, α-smooth muscle actin (α-SMA), type Ι collagen (collagen 1), fibronectin, TGF-β1, IL-11, and IL-11 receptor α1 (Rα1) compared with those of WT mice; protein levels of collagen 1, α-SMA, mature TGF-β1 and IL-11 and mRNA levels of *Col1a1*, *Col1a2*, *ACTA2*, *PAI-1*, *TGF-β1*, *MMP9*, *MMP12*, *IL-11*, and *IL-11Rα1* were also significantly decreased in *Bmi-1*^*−/−*^ mice compared with those of WT mice. Significant decreases were observed in the percentage of SFTPC-labeled AT2 cells and the expression of SFTPC at the mRNA and protein levels (Figs. 3 and 4a–d). We also assessed whether senescent fibroblasts produced profibrotic factors. The percentage of double-positive areas for vimentin & TGF-β1, vimentin & IL-11, vimentin & p16, ER-TR7 & p53, and ER-TR7 & p16 were increased in *Bmi-1*^*−/−*^ mice compared with those of WT mice (Fig. S2). IL-11Rα1 was expressed in SFTPC-labeled AT2 cells (Fig. 3a), suggesting that TGF-β1 and IL-11 affect the EMT of AT2 cells. NAC treatment decreased senescent fibroblasts and profibrotic factors in interstitial fibroblasts and increased SFTPC expression by AT2 cells more than that of *p16* deletion. Expression of *Col1a1*, *Col1a2*, *TGF-β1*, and *MMP9* at the mRNA level also increased (Figs. 3, 4a–d, and S2). These results suggest that NAC treatment was better than *p16* deletion at preventing SAPF by inhibiting TGF-β1 and IL-11 and maintaining the proportion and nature of AT2 cells.

### NAC-induced inhibition of TIME signaling-mediated SAPF in *Bmi-1*^*−/−*^ mice vs. *Bmi-1*^*−/−*^*p16*^*−/−*^ mice

To determine whether SAPF was mediated by TIME signaling and ameliorated by NAC treatment or *p16* deletion, the lungs were examined for proteins related to signaling. In *Bmi-1*^*−/−*^ mice, significant increases were observed in the expression of the TGF-β1-Smad-signaling proteins, including mature TGF-β1, TGF-β receptor-type 2 (RII), Smad2, pSmad2 (Ser465/467), and pSmad2/3 (Ser423/425), compared with those of WT mice. Significant increases in expression were observed in the IL-11/MEK/ERK/RSK signaling proteins IL-11, IL-11Rα, MEK1/2, pMEK1/2 (Ser217/221), elF4E, p-elF4E (Ser209), RSK and p-RSK (Ser380), and the ratio of pERK1/2 (Thr202/Tyr204) to ERK1/2. NAC treatment decreased the signaling proteins more than *p16* deletion (Fig. 4a–d). To determine whether the increases in pulmonary TGF-β1 and IL-11 proteins were affected by other organs or tissues in *Bmi-1*^*−/−*^ mice, serum TGF-β1 and IL-11 were detected by ELISA. Levels of TGF-β1 and IL-11 in serum samples were not consistent with levels in lung tissue, and serum IL-11 protein levels were not altered among the four groups of mice. These results demonstrate that pulmonary fibrosis caused by TGF-β1 and IL-11 mainly occurred in cells inside lung tissue (Fig. S3).

To investigate whether IL-11 mediates the profibrotic effect of TGF-β1 by activating MEK/ERK/RSK signaling, pulmonary fibroblasts from WT mice were treated with TGF-β1, TGF-β1 plus anti-IL-11 antibody, IL-11, or IL-11 plus the MEK inhibitor PD98059. The expression of IL-11, p16 and collagen 1 significantly increased with TGF-β1 or IL-11 treatment compared with that of treatment with vehicle. Anti-IL-11 or PD98059 decreased the expression of IL-11, p16, and collagen 1 in TGF-β1- or IL-11-treated cells, suggesting that TGF-β1 or IL-11 treatment promotes fibroblast senescence. The alteration in IL-11 protein expression from these treatments was consistent with the results of p16 protein expression (Fig. 4e–h), suggesting that the profibrotic effect of IL-11 might be closely related to cell senescence.

### Inhibition of cell senescence and TIME signals in pulmonary fibroblasts by NAC in *Bmi-1*^*−/−*^ vs. *Bmi-1*^*−/−*^*p16*^*−/−*^ mice

**Fig. 5:**
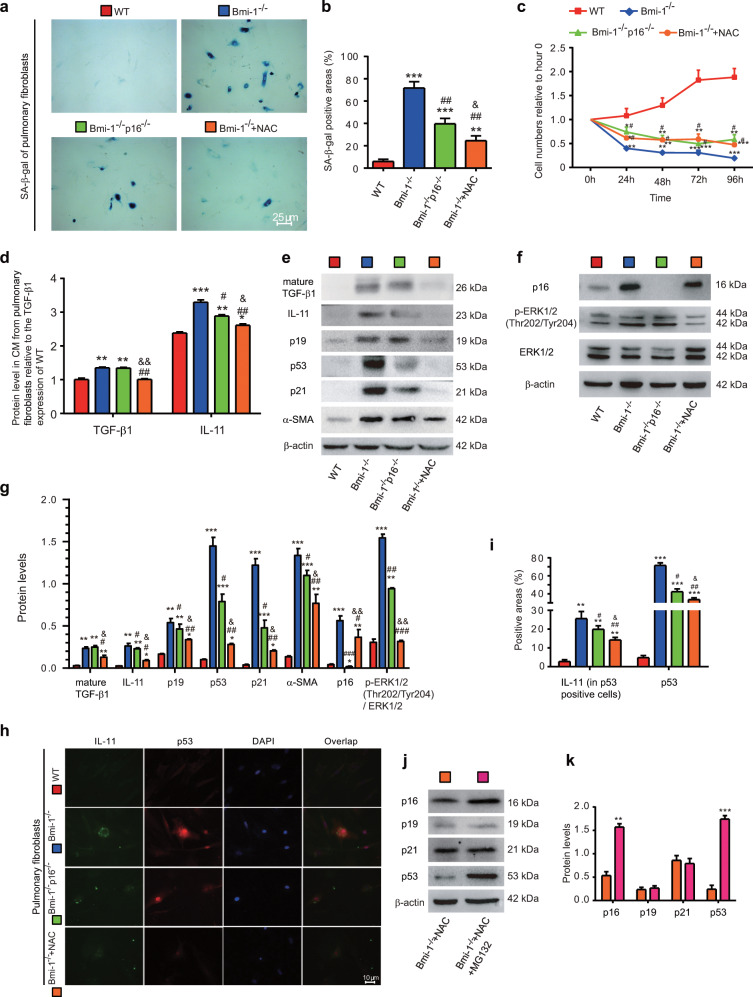
NAC treatment inhibited cell senescence and TIME signaling in *Bmi-1*^*−/−*^ more than in *Bmi-1*^*−/−*^*p16*^*−/−*^ pulmonary fibroblasts. Pulmonary fibroblasts from 7-week-old WT, *Bmi-1*^*−/−*^, *Bmi-1*^−/−^*p16*^−/−^, and NAC-treated *Bmi-1*^*−/−*^ (*Bmi-1*^*−/−*^ + NAC) mice. **a** Representative micrographs of cells stained cytochemically for SA-β-gal. **b** Percentage of SA-β-gal-positive areas relative to the total areas. **c** Third-passage fibroblast proliferation was determined by CCK-8 assays and spectrophotometry at 450 nm. Cell number was determined at the indicated hours relative to the cell number at hour 0. **d** TGF-β1 and IL-11 concentrations were detected in conditioned medium (CM) by ELISA and assessed by densitometric analysis relative to that of WT CM. **e**, **f** Western blots for mature TGF-β1, IL-11, p19, p53, p21, α-SMA, p16, pERK1/2 (Thr202/Tyr204), and ERK1/2. β-actin was used as the loading control. **g** Protein expression relative to β-actin was assessed by densitometric analysis. **h** Representative micrographs showing immunofluorescence for IL-11 and p53, with DAPI staining the nuclei. **i** Percentage of IL-11-positive areas (in p53-positive cells) or p53-positive areas relative to the total area assessed. Six biological replicates were used per experiment. Values are the means ± SEM of six determinations. *P < 0.05, **P < 0.01, ***P < 0.001 compared with the WT group; ^#^*P* < 0.05, ^##^*P* < 0.01, ^###^*P* < 0.001 compared with the *Bmi-1*^*−/−*^ group; ^&^*P* < 0.05, ^&&^*P* < 0.01 compared with the *Bmi-1*^−/−^*p16*^−/−^ group. **j** Western blots of pulmonary fibroblasts from the NAC (1 mm, 48 h)-treated *Bmi-1*^*−/−*^ (*Bmi-1*^*−/−*^ + NAC) group and the NAC (1 mm, 48 h) & MG132 (5 μm, 48 h)-treated *Bmi-1*^*−/−*^ (*Bmi-1*^*−/−*^ + NAC + MG132) group. **k** Protein expression relative to β-actin was assessed by densitometric analysis. Six biological replicates were used per experiment. Values are the means ± SEM of six determinations. **P < 0.01, ***P < 0.001 compared with the NAC-treated *Bmi-1*^*−/−*^ group.

We investigated whether cell senescence and activation of TIME signals in pulmonary fibroblasts were responsible for initiating interstitial fibrosis and were ameliorated by NAC treatment or *p16* deletion. Cell senescence-, proliferation-, and TIME signaling-related proteins were examined. In *Bmi-1*-null pulmonary fibroblasts, the percentages of SA-β-gal-positive cells or areas significantly increased compared with those of WT cells. In addition, the number of cells decreased owing to cell death. Significant increases were observed in the expression of secreted TGF-β1 and IL-11. In addition, obvious increases were observed in the protein expression of mature TGF-β1, IL-11, p19, p53, p21, α-SMA, and p16, the ratio of pERK1/2 (Thr202/Tyr204) to ERK1/2, and the expression of IL-11 by p53-labeled-senescent cells. NAC treatment delayed cell senescence, maintained cell numbers, and decreased TIME signaling-related proteins more than those of *p16* deletion but did not affect cell numbers (Fig. 5a–i). These results suggest that activation of TIME signaling and retention of TGF-β1 and IL-11 in senescent pulmonary fibroblasts promotes the EMT of nearby AT2 cells.

To determine whether NAC mediates protein degradation of the cyclin-dependent kinase inhibitors p16, p19, p21, and p53 via ubiquitin-proteasome pathways, the proteasome inhibitor MG132 was used to inhibit proteasomes in *Bmi-1*^*−/−*^ fibroblasts treated with NAC. Compared with the levels in *Bmi-1*^*−/−*^ pulmonary fibroblasts treated with NAC, p16, and p53 significantly increased. In *Bmi-1*^*−/−*^ fibroblasts treated with NAC and MG132, p19 and p21 were not altered, suggesting that NAC participates in ubiquitin-proteasome degradation of p16 and p53 (Fig. 5j, k).

### NAC-induced inhibition of cell senescence and TIME signaling mediates the EMT in AT2 cells in *Bmi-1*^*−/−*^ vs. *Bmi-1*^*−/−*^*p16*^*−/−*^ mice

**Fig. 6:**
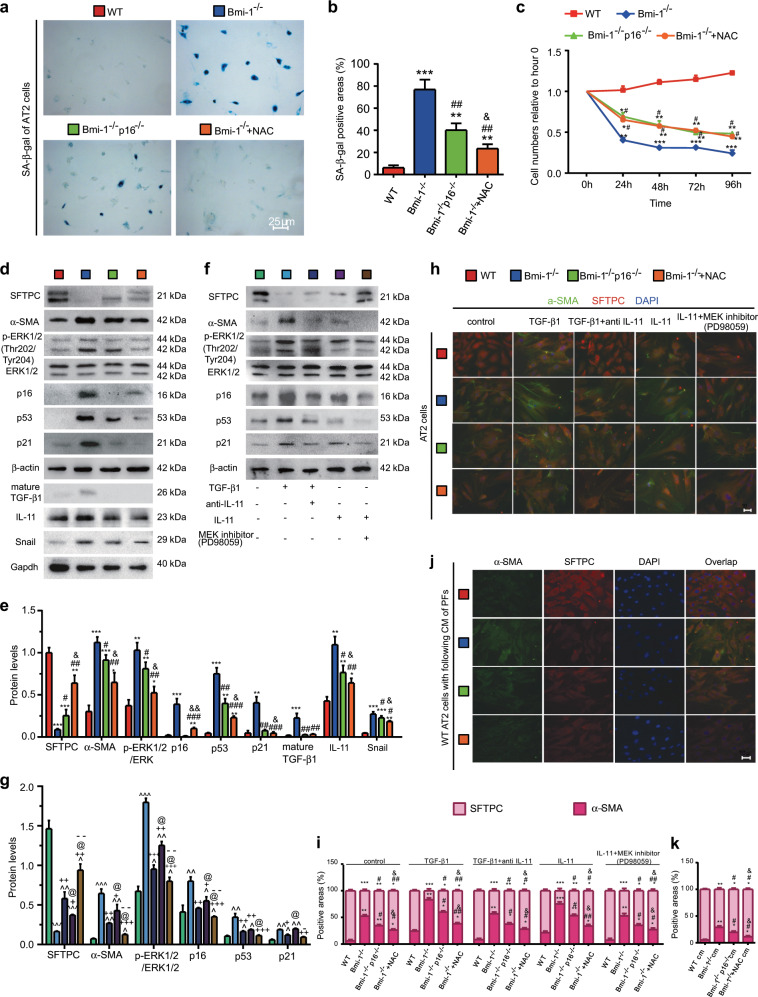
NAC treatment inhibited cell senescence, and TIME signaling mediated the EMT in *Bmi-1*^*−/−*^ cells more than in *Bmi-1*^*−/−*^*p16*^*−/−*^ AT2 cells. Pulmonary AT2 cells from 7-week-old WT, *Bmi-1*^*−/−*^, *Bmi-1*^−/−^*p16*^−/−^, and NAC-treated *Bmi-1*^*−/−*^ (*Bmi-1*^*−/−*^ + NAC) mice. **a** Representative micrographs of cells stained cytochemically for SA-β-gal. **b** Percentage of SA-β-gal-positive areas relative to the total. **c** Second-passage AT2 cell proliferation were determined by CCK-8 assays, and spectrophotometry at 450 nm. Cell number was determined at the indicated hours relative to the cell number at hour 0. **d** Western blots for SFTPC, α-SMA, pERK1/2 (Thr202/Tyr204), ERK1/2, p16, p53, p21, mature TGF-β1, IL-11, and Snail. β-actin was used as the loading control. **e** Protein expression relative to β-actin was assessed by densitometric analysis. **f** Pulmonary AT2 cells were treated with TGF-β1 (5 ng/ml, 48 h), TGF-β1 (5 ng/ml, 48 h), and anti-IL-11 antibody (2 μg/ml, 48 h), IL-11 (5 ng/ml, 48 h), or IL-11 (5 ng/ml, 48 h) and the MEK inhibitor (PD98059) (10 μm, 48 h). Western blots for SFTPC, α-SMA, pERK1/2 (Thr202/Tyr204), ERK1/2, p16, p53, and p21. β-actin was used as the loading control. **g** Protein expression relative to β-actin was assessed by densitometric analysis. **h** Representative micrographs of cells immunofluorescently stained for α-SMA and SFTPC, with DAPI staining the nuclei. **i** The percentage of α-SMA- or SFTPC-positive areas was relative to the total. **j** WT AT2 cells treated with CM from third-passage pulmonary fibroblasts from the WT, *Bmi-1*^*−/−*^, *Bmi-1*^−/−^*p16*^−/−^ or NAC-treated *Bmi-1*^*−/−*^ (*Bmi-1*^*−/−*^ + NAC) groups. **k** The percentage of α-SMA- or SFTPC-positive areas relative to the total. Six biological replicates were used per experiment. Values are the means ± SEM of six determinations. *P < 0.05, **P < 0.01, ***P < 0.001 compared with the WT group; ^#^*P* < 0.05, ^##^*P* < 0.01, ^###^*P* < 0.001 compared with the *Bmi-1*^*−/−*^ group; ^&^*P* < 0.05, ^&&^*P* < 0.01 compared with the *Bmi-1*^−/−^*p16*^−/−^ group. ^^^*P* < 0.05, ^^^^*P* < 0.01, ^^^^^*P* < 0.001 compared with the vehicle group; ^+^*P* < 0.05, ^++^*P* < 0.01, ^+++^*P* < 0.001 compared with the TGF-β1 treatment group; ^@^*P* < 0.05 compared with the TGF-β1 and anti-IL-11 antibody treatment group; ^−^*P* < 0.05, ^−−^P < 0.01 compared with the IL-11 treatment group.

To determine whether NAC treatment or *p16* deletion ameliorates cell senescence- and TIME signaling-mediated EMT in AT2 cells, cell senescence and EMT-related proteins were detected. In *Bmi-1*-null AT2 cells, the percentages of SA-β-gal-positive cells or areas significantly increased compared with those of WT cells (Fig. 6a, b). In addition, the number of cells decreased owing to cell death (Fig. 6c). Significant increases were observed in the protein expression of α-SMA, p16, p53, p21, mature TGF-β1, IL-11, and Snail and the ratio of pERK1/2 (Thr202/Tyr204) to ERK1/2, whereas a significant decrease was observed in the expression of SFTPC (Fig. 6d, e). Significant increases were also observed in the expression of p53, TGF-β RII, and IL-11Rα1. TGF-β RII and IL-11Rα1 were mainly located in p53-labeled-senescent cells (Fig. S4). Treatment with vehicle, TGF-β1, TGF-β1 plus anti-IL-11, IL-11, or IL-11 plus PD98059 resulted in significant increases in the expression of α-SMA, p16, p53, and p21 and the ratio of pERK1/2 (Thr202/Tyr204) to ERK1/2, whereas a significant decrease was observed in the expression of SFTPC. The percentages of α-SMA-positive cells or areas significantly increased, whereas the percentages of SFTPC-positive cells or areas significantly decreased in *Bmi-1*-null AT2 cells compared with those of WT cells (Fig. 6f–i). NAC treatment maintained cell numbers and ameliorated cell senescence, expression of p16, p53, p21, TGF-β RII, and IL-11Rα1, and MEK/ERK signals mediated the EMT more than that of *p16* deletion, although cell numbers were unchanged. NAC treatment and *p16* deletion decreased the expression of mature TGF-β1, IL-11, and Snail in AT2 cells. Moreover, NAC treatment decreased the expression of IL-11 and Snail more than that of *p16* deletion (Fig. 6a–i and S4). Significant increases were observed in the expression of α-SMA, p16, p53, and p21 and the ratio of pERK1/2 (Thr202/Tyr204) to ERK1/2 in the WT, *Bmi-1*^−/−^, *Bmi-1*^−/−^*p16*^−/−^, and NAC-treated *Bmi-1*^−/−^ groups compared with that of the vehicle, whereas a significant decrease was observed in the expression of SFTPC. The percentages of α-SMA-positive cells or areas significantly increased and the percentages of SFTPC-positive cells or areas significantly decreased with TGF-β1 or IL-11 treatment in the WT, *Bmi-1*^−/−^, *Bmi-1*^−/−^*p16*^−/−^, and NAC-treated *Bmi-1*^−/−^ groups compared to those of the vehicle. The EMT and cell senescence of AT2 cells induced by TGF-β1 or IL-11 treatment was ameliorated by anti-IL-11 or PD98059 treatment (Fig. 6f–i), demonstrating that TIME signals mediate the EMT and cell senescence in AT2 cells.

To determine whether retention of TGF-β1 and IL-11 in senescent pulmonary fibroblasts promoted the EMT of AT2 cells, cells from WT mice were treated with conditioned medium (CM) from pulmonary fibroblasts from WT, *Bmi-1*^−/−^, *Bmi-1*^−/−^*p16*^−/−^, or NAC-treated *Bmi-1*^−/−^ mice. In CM-treated *Bmi-1*^*−/−*^ cells, the percentages of α-SMA-positive areas were significantly increased, whereas the percentages of SFTPC-positive areas were significantly decreased, compared with those of the WT cells. CM from NAC-treated cells ameliorated these reactions more than those of *p16* deletion (Fig. 6j, k). These results demonstrated that overproduction of TGF-β1 and IL-11 in senescent pulmonary fibroblasts promoted the EMT of AT2 cells.

### Inhibition of pERK1/2 (Thr202/Tyr204) translocation from the cytoplasm to the nucleus by p16-binding activated MEK/ERK signaling

**Fig. 7:**
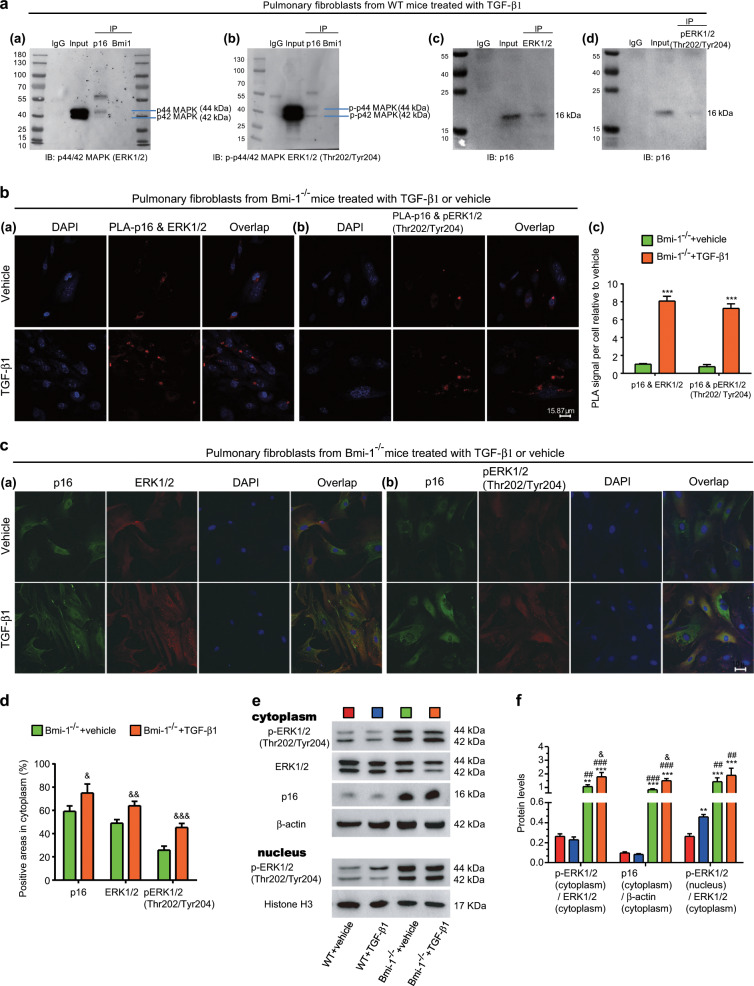
Inhibition of pERK1/2 (Thr202/Tyr204) translocation from the cytoplasm to the nucleus by p16 binding. **a** Pulmonary fibroblasts from WT mice were treated with TGF-β1 (5 ng/ml, 48 h), and cytoplasmic proteins were extracted for p16, Bmi-1, ERK1/2, or pERK1/2 (Thr202/Tyr204) coimmunoprecipitation. Western blots detecting ERK1/2, pERK1/2(Thr202/Tyr204) or p16. Pulmonary fibroblasts from *Bmi-1*^*−/−*^ mice treated with TGF-β1 (5 ng/ml, 48 h) or vehicle. **b** Representative micrographs of Duolink proximity ligation assays (PLAs) for the interaction between p16 and ERK1/2 or p16 and pERK1/2 (Thr202/Tyr204), with DAPI staining the nuclei. PLA signal per cell relative to the vehicle. **c** Representative micrographs of cells immunofluorescently stained for p16 and ERK1/2 or p16 and pERK1/2 (Thr202/Tyr204), with DAPI staining the nuclei. **d** The percentage of areas positive for p16, ERK1/2, or pERK1/2 (Thr202/Tyr204) in the cytoplasm relative to the total. **e** Western blots of ERK1/2, pERK1/2 (Thr202/Tyr204), and p16 in the cytoplasm and pERK1/2 (Thr202/Tyr204) in the nucleus of pulmonary fibroblasts from WT and *Bmi-1*^−/−^ mice treated with vehicle or TGF-β1. **f** Protein expression relative to β-actin in the cytoplasm and histone H3 in the nucleus was assessed by densitometric analysis. Three biological replicates were used per experiment. Values are the means ± SEM of six determinations. **P < 0.01, ***P < 0.001 compared with the vehicle-treated WT (Vehicle + WT) group; ^##^*P* < 0.01, ^###^*P* < 0.001 compared with the TGF-β1-treated WT (TGF-β1 + WT) group; ^&^*P* < 0.05, ^&&^*P* < 0.01, ^&&&^*P* < 0.001 compared with the vehicle-treated *Bmi-1*^*−/−*^ (Vehicle + *Bmi-1*^*−/−*^) group.

To investigate whether p16 inhibited translocation of pERK1/2 (Thr202/Tyr204) from the cytoplasm to nucleus, pulmonary fibroblasts from WT mice were treated with TGF-β1 and proteins were extracted for p16 or Bmi-1 coimmunoprecipitation analysis. P16 interacted with ERK1/2 or pERK1/2 (Thr202/Tyr204) (Fig. 7a). We further used Duolink PLA in situ to confirm the interaction of p16 and ERK1/2 or p16 and pERK1/2 (Thr202/Tyr204) in the cytoplasm (Fig. 7b). Pulmonary fibroblasts from *Bmi-1*^−/−^ mice were treated with TGF-β1 and examined for the colocalization of p16 and ERK1/2 or p16 and pERK1/2 (Thr202/Tyr204). In the TGF-β1-treatment group, the expression of p16, ERK1/2, and pERK1/2 (Thr202/Tyr204) in the cytoplasm significantly increased compared with that of the vehicle group. In addition, p16 overlapped with ERK1/2 or pERK1/2 (Thr202/Tyr204) in the cytoplasm (Fig. 7c, d). We also detected ERK1/2, pERK1/2 (Thr202/Tyr204) and p16 in the cytoplasm and pERK1/2 (Thr202/Tyr204) in the nucleus of pulmonary fibroblasts WT and *Bmi-1*^−/−^ mice treated with TGF-β1 or vehicle from. In the cytoplasm, TGF-β1 treatment significantly increased p16 expression and the ratio of pERK1/2 (Thr202/Tyr204) to ERK1/2 in *Bmi-1*^−/−^ fibroblasts. TGF-β1 treatment did not alter these factors in WT fibroblasts. In the nucleus, TGF-β1 treatment increased pERK1/2 (Thr202/Tyr204) in WT fibroblasts, whereas this treatment did not alter this protein in *Bmi-1*^−/−^ fibroblasts (Fig. 7e, f). These results demonstrated that *p16* deletion or downregulation promoted translocation of pERK1/2 (Thr202/Tyr204) from the cytoplasm to the nucleus to downregulate MEK/ERK signaling.

**Fig. 8:**
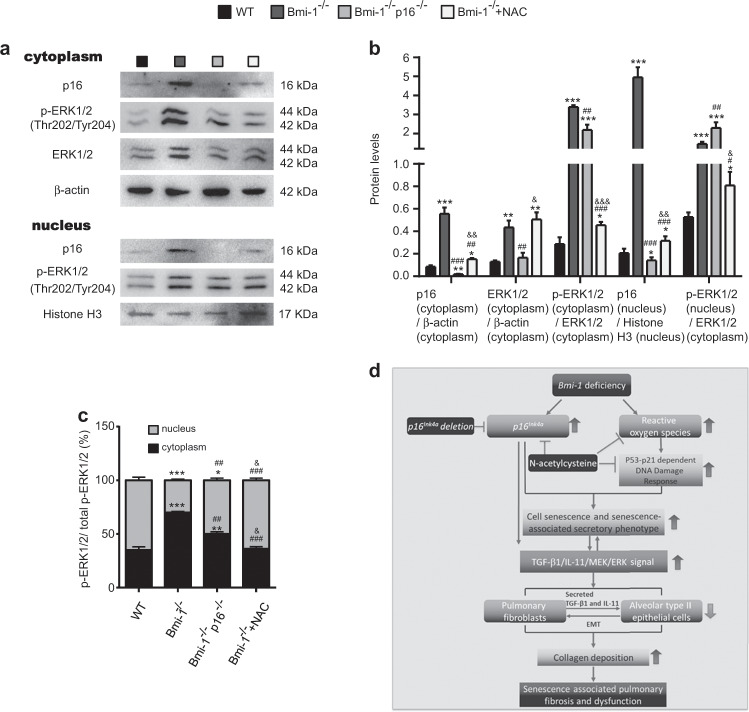
*P16* deletion or downregulation promoted translocation of pERK1/2 (Thr202/Tyr204) from the cytoplasm to the nucleus. **a** Western blots of p16, pERK1/2 (Thr202/Tyr204), and ERK1/2 in the cytoplasm and p16 and pERK1/2 (Thr202/Tyr204) in the nucleus of fibroblasts from WT, *Bmi-1*^*−/−*^, *Bmi-1*^−/−^*p16*^−/−^, or NAC-treated *Bmi-1*^*−/−*^ (*Bmi-1*^*−/−*^ + NAC) mice. **b** Protein expression relative to β-actin in the cytoplasm and histone H3 in the nucleus was assessed by densitometric analysis. **c** pERK1/2 (Thr202/Tyr204) protein levels relative to total pERK1/2 (Thr202/Tyr204) protein levels in the cytoplasm and nucleus. Three biological replicates were used per experiment. Values are the means ± SEM of six determinations. *P < 0.05, **P < 0.01, ***P < 0.001 compared with the WT group; ^#^*P* < 0.05, ^##^*P* < 0.01, ^###^*P* < 0.001 compared with the *Bmi-1*^*−/−*^ group; ^&^*P* < 0.05, ^&&^*P* < 0.01, ^&&&^*P* < 0.001 compared with the *Bmi-1*^−/−^*p16*^−/−^ group. **d** Graphical Abstract In *Bmi-1* null mice, upregulated TGF-β1/IL-11/MEK/ERK (TIME) signaling contributed to senescence-associated pulmonary fibrosis (SAPF) and dysfunction by promoting cell senescence and stimulating TGF-β1 and IL-11 secretion and collagen 1 production in aging pulmonary fibroblasts and the epithelial-to-mesenchymal transition (EMT) in aging alveolar type II epithelial cells. NAC treatment ameliorated pulmonary dysfunction and SAPF by downregulating TIME signaling more than that of *p16* deletion by inhibiting oxidative stress and DNA damage and promoting ubiquitin-proteasome degradation of p16 and p53.

We directly compared the amount of pERK1/2 (Thr202/Tyr204) in the cytoplasm and nucleus among fibroblasts from WT, *Bmi-1*^−/−^, *Bmi-1*^−/−^*p16*^−/−^, and NAC-treated *Bmi-1*^−/−^ mice. Compared with expression in the *Bmi-1*-deficient group, the expression of p16, ERK1/2, and pERK1/2 (Thr202/Tyr204) in the cytoplasm significantly decreased in the *p16* and *Bmi-1* double-knockout group, the expression of pERK1/2 (Thr202/Tyr204) in the nucleus was obviously increased in the *p16* and *Bmi-1* double-knockout group and the ratio of pERK1/2 (Thr202/Tyr204) in the nucleus to total pERK1/2 (Thr202/Tyr204) was obviously increased (Fig. 8a–c). Therefore, *p16* deletion or downregulation promoted translocation of pERK1/2 (Thr202/Tyr204) from the cytoplasm to the nucleus.

### TIME signals may also mediate human SAPF

To determine whether human SAPF was caused by cell senescence and mediated by TIME signaling, collagen 1, α-SMA, p16, p53, p21, TGF-β1, IL-11, and pERK1/2 (Thr202/Tyr204) were detected in human pulmonary tissue by using ELISA and were analyzed for correlations. Collagen 1 positively correlated with p16, IL-11, pERK1/2 (Thr202/Tyr204), TGF-β1, p21, p53, and α-SMA; p16 positively correlated with IL-11, pERK1/2 (Thr202/Tyr204), TGF-β1, p21, p53, and α-SMA; IL-11 positively correlated with pERK1/2 (Thr202/Tyr204), TGF-β1, p21, p53, and α-SMA; pERK1/2 (Thr202/Tyr204) positively correlated with p21, p53, and α-SMA; p21 positively correlated with p53 and α-SMA; and p53 positively correlated with α-SMA (Fig. S5a–x). These results indicate that the accumulation of collagen 1 and α-SMA in the lungs accompanied by cell senescence may be mediated by TIME signaling.

To confirm whether the SA-β-gal activity and α-SMA-positive areas in human pulmonary tissues correlated with p16 protein levels, we made frozen tissue sections of human pulmonary tissues, detected the SA-β-gal activity and α-SMA immunohistological expression, and found that the percentages of SA-β-gal-positive and α-SMA-positive areas were increased in conjunction with the p16 protein levels in human pulmonary tissues (Fig. S5y–z).

## Discussion

This study demonstrated that in a SIPS model of *Bmi-1* deficiency, upregulated TIME signals contributed to SAPF and dysfunction by promoting cell senescence and stimulating TGF-β1 and IL-11 secretion and collagen 1 production in aging pulmonary fibroblasts and the EMT in aging AT2 cells (Fig. 8d). NAC treatment prolonged the lifespan and rescued alterations by downregulating TIME signals more than that of *p16* deletion owing to its inhibition of oxidative stress and DNA damage and promotion of ubiquitin-proteasome degradation of p16 and p53. Cytoplasmic p16 retention upregulated MEK/ERK signals by inhibiting translocation of pERK1/2 (Thr202/Tyr204) from the cytoplasm to the nucleus in senescent fibroblasts. The accumulation of collagen 1 and α-SMA in human lungs accompanied by cell senescence was mediated by TIME signaling.

Previous clinical observations demonstrated that IPF was associated with aging, fibrosing interstitial pneumonia and chronic, progressive pulmonary dysfunction. These factors lead to symptoms including frailty, fatigue, weight loss, and shortened walking distance and lifespan^1,2,29,30^. The results with *Bmi-1*-deficient mice are consistent with these typical symptoms of IPF^4,5,12,23^. This study evaluated pulmonary functional and histological alterations to determine if *Bmi-1*-deficient mice showed senescence-associated fibrosing interstitial pneumonia. We found that *Bmi-1* deficiency caused ventilatory resistance and poor ventilatory compliance. Accompanied by accumulated cell senescence, cycle arrest, ROS, and DNA damage, many inflammatory cells and proinflammatory factors, including IL-1β, IL-6, and TNF-α, were abnormally accumulated in interstitial spaces around the walls of the lung air sacs (alveoli), blood vessels and small airways in *Bmi-1*-deficient mice. A previous study found that owing to a profound effect in the lung microenvironment of cell senescence and SASP, the secretion of a variety of mediators contributed to the development and perpetuation of fibrosis^2^. We found significant increases in interstitial fibers, collagen 1, fibronectin, α-SMA-labeled myofibroblasts, and the profibrotic factors TGF-β1 and IL-11 and a significant decrease in SFTPC-labeled AT2 cells. Thus, *Bmi-1* deficiency caused SAPF in the same manner as IPF.

Recent observations revealed that IL-11, the crucial fibrosis gene acting downstream of TGF-β1, also activated ERK in cardiac fibroblasts. Both TGFβ-1 and IL-11 require ERK to induce profibrotic phenotypes^6^. *IL-11* is upregulated by 100-fold in fibroblasts from patients with IPF, suggesting this gene is critical in human pulmonary fibrosis^8^. Therefore, we examined whether pulmonary fibrosis caused by *Bmi-1* deficiency was associated with activation of TIME signals. We found that *Bmi-1* deficiency significantly activated TIME signaling leading to SAPF, with TGF-β1 and IL-11 mainly produced by inner pulmonary cells but not in the serum. SAPF is characterized by the accumulation of SA-β-gal-positive-senescent fibroblasts, and the SASP is indeed fibrogenic^1^. Our results demonstrated that *Bmi-1* deficiency caused the senescence of pulmonary fibroblasts that secreted TGF-β1 and IL-11 and produced collagen 1. IL-11 mediated the profibrotic effect of TGF-β1 and further promoted aging in senescent pulmonary fibroblasts by activating MEK/ERK signals. This process was inhibited by anti-IL-11 or the MEK inhibitor PD98059. Consistent with this result is a previous observation that PD98059 reduces lung injury and inflammation and suppresses the development of lung fibrosis in a bleomycin model^31^. Thus, IL-11/MEK/ERK signaling may be a therapeutic target for preventing SAPF.

Primary human AT2 cells undergo a TGFβ-dependent EMT phenotypic change and acquire the potential to produce collagen 1^9^. Several lines of evidence suggest that the radiation-induced EMT of AT2 cells is important in pulmonary fibrosis that is mediated by the TGF-β and ERK/glycogen synthase kinase 3β (GSK3β) pathways^32,33^. We observed that TGF-β RII and IL-11Rα1 expression was higher in senescent fibroblasts and in senescent AT2 cells compared to that of normal cells. We examined whether senescent fibroblasts, through the SASP, affected the TIME signaling-mediated EMT of adjacent senescent AT2 cells. We found that *Bmi-1* deficiency also induced senescence of AT2 cells that were prone to EMT induction by CM, including secreted TGF-β1 and IL-11 from *Bmi-1*-null-senescent fibroblasts. IL-11 mediated the TGF-β1 effect of driving cell senescence and the EMT of AT2 cells by activating MEK/ERK signaling. This activation was inhibited by anti-IL-11 or PD98059. Therefore, overproduction of TGF-β1 and IL-11 in senescent pulmonary fibroblasts promoted the EMT and cell senescence of nearby AT2 cells to exacerbate fibrosis.

Bmi-1 normally simultaneously represses the Ink4a/Arf locus, leading to reduced *p16* and *p19* expression and modulating mitochondrial function and redox balance to reduce ROS levels and suppress DNA damage reaction pathway activation to limit aging^4,12^. Whether the major downstream mediator of Bmi-1 is p16 or ROS is unknown, as is their crosstalk in SAPF. A study demonstrated that p16-positive senescent fibroblasts were selectively killed by a senolytic cocktail that decreased the profibrotic effects of the SASP and improved pulmonary function and physical health in a bleomycin-injury IPF model^1^. Oxidative stress, an important molecular mechanism underlying fibrosis in pulmonary fibrosis, increases TGF-β1-induced fibrosis in part by activating latent TGF-β1. This activation, in part by inducing DNA damage, stimulates ROS production, leading to oxidative stress and results in a vicious profibrogenic circle^34,35^. NAC, a glutathione (GSH) precursor, increases pulmonary GSH levels and attenuates bleomycin-induced fibrosis^34^. We compared the effects of NAC treatment and *p16* deletion on ameliorating aging-associated pulmonary fibrosis in *Bmi-1*-deficient mice. Our results indicated that NAC treatment was better than *p16* deletion at prolonging the lifespan and downregulating TIME signaling that contribute to SAPF and dysfunction by promoting cell senescence and stimulating TGF-β1 and IL-11 secretion and collagen 1 production in aging pulmonary fibroblasts and the EMT in aging AT2 cells. We also found that NAC treatment was better than *p16* deletion at decreasing the expression of p16 protein but not mRNA and exerted better anti-aging effects by inhibiting the expression of p19, p53, p21, and 8-OHdG in the lungs. NAC-mediated protein degradation of p16 and p53 via ubiquitin-proteasome pathways. Several lines of evidence demonstrate that the deletion of *p16* in *Bmi-1*-deficient mice did not alter ROS levels, suggesting that the oxidative stress that occurs in *Bmi-1*-deficient mice does not result from the *p16* senescence pathway upregulation^4,12^. A previous study showed that increased ROS promotes dissociation of Bmi-1 from chromatin and upregulates *p16* transcription by activating MEK/ERK signaling and causing cell senescence. NAC treatment significantly downregulates MEK/ERK signaling to prevent cellular senescence^36^. Our results showed that NAC treatment significantly inhibited TGF-β1/IL-11/MEK/ERK signaling to prevent pulmonary fibrosis in *Bmi-1*^−/−^ mice. Thus, ROS might be a major downstream mediator by which Bmi-1 deficiency regulates SAPF. However, the exact regulatory mechanism of antioxidants on TIME signaling molecules remains to be investigated.

A previous observation found that increased expression of p-ERK1/2 (Thr202/Tyr204) and retention in the cytoplasm further activates MEK/ERK signaling during cell senescence^37^. The constitutive induction of p-ERK1/2 (Thr202/Tyr204) has been shown in both replicative senescence and oncogenic Ras-induced premature senescence cells, which was denoted as “senescence-associated persistent induction of p-ERK1/2 (SA-p-ERK1/2)”^38^. Several lines of evidence suggest that depending on the integrity of the senescence program controlled by the cell cycle inhibitors p16, p21, and p53, cellular senescence is induced by the constitutively activated MEK/ERK signaling pathway in different tissues and cell types, including mouse embryos, fibroblasts, and intestinal epithelial cells^39–41^. It has been reported that the regulation of *p15 (INK4b)* and *p16 (INK4a)* mRNA and protein levels is mediated by the ERK signaling pathway^42^. A high level of activation of the MEK–ERK pathway in fetal hepatoblasts induces the accumulation of p16 or p19^43^. Therefore, we explored how MEK/ERK induced senescence. The mechanism of the relationship between MEK/ERK and cyclin-dependent kinase inhibitors is still unclear. In the cytoplasm of old cells, a large amount of SA-p-ERK1/2 protein is bound and sequestered and cannot translocate into the nucleus to regulate cell cycle, proliferation, differentiation, and senescence^38,44^. Therefore, we focused on the interaction between p16 and ERK/pERK (Thr202/Tyr204), p21 and ERK/pERK (Thr202/Tyr204), and p53 and ERK/pERK (Thr202/Tyr204). We found that p16 only interacted with ERK/pERK (Thr202/Tyr204). We further used a PLA to confirm the interaction between p16 and ERK1/2 or p16 and pERK1/2 (Thr202/Tyr204) in the cytoplasm. As an important messenger of extracellular and intracellular signals, ERK1/2 has a critical role in cellular outcomes involving numerous substrates, regulators and scaffolding proteins, and translocation of ERK1/2 from the cytoplasm to the nucleus is essential for regulation of the cell cycle, proliferation, differentiation, and senescence^44^. Therefore, we observed the translocation of pERK (Thr202/Tyr204). Our study found that p16 directly combined with cytoplasmic p-ERK1/2 (Thr202/Tyr204) to prevent translocation to the nucleus and further activate MEK/ERK signals, in addition to indirectly promoting SASP in senescent fibroblasts. Previous observations demonstrated that the N-terminal portion of p16 (residues 1–80) bound to the N-terminal region of JNK1 (also known as MAPK8) (residues 1–60) or the N-terminal region of JNK3 (also known as MAPK10) (residues 75–100), which contain the glycine-rich site^45^. In this study, we found that p16 bound to the ERK1/2 protein (also known as MAPK3/1). We hypothesized that the same domain might be involved in the interaction between p16 and MAPK1/3 or MAPK8/10. Analysis with https://www.uniprot.org/align showed that an extremely similar domain existed between MAPK3 (from 44th residue to 304th residue) and MAPK8 (from 26th residue to 321st residue), an extremely similar domain existed between MAPK1 (from 26th residue to 313th residue) and MAPK8 (from 26th residue to 313th residue), an extremely similar domain existed between MAPK3 (from 42nd residue to 330th residue) and MAPK10 (from 64th residue to 359th residue), and an extremely similar domain also existed between MAPK1 (from 25th residue to 313th residue) and MAPK10 (from 64th residue to 359th residue) (Supplementary Information 4). Using https://www.uniprot.org/align, we further found that MAPK1/3 had the same common conserved regions and conserved amino residues in the common interacting regions as MAPK8/10 (Supplementary Information 5). Thus, we thought that MAPK1/3 might bind to p16 through the same domain as MAPK8/10. In a future study, we will further determine the effects of MAPK1/3 anchored by p16 in the cytoplasm in aging cells and confirm the binding domain between p16 and MAPK1/3.

The literature has evidence suggesting that the expression of fibrogenic SASP factors, including *Col1a1*, *PAI1*, and *MMP10*, robustly correlates with *p16* transcriptional activation in bleomycin-induced fibrosis^1^. In human pulmonary tissue of different ages, our findings indicated that the accumulation of collagen 1 and α-SMA in the lungs accompanied by cell senescence was mediated by TIME signaling. It remains to be further determined whether these signals mediate human SAPF. Because age does not completely determine the degree of cellular aging, we chose the p16 protein levels in human pulmonary tissues as the main aging evaluation marker. As a biomarker, effector and regulator of the aging program, p16 is also a premier indicator of the presence of senescent cells. It is often transcriptionally activated in cells undergoing irreversible senescence, which leads to aging-associated impaired function and regenerative capacity^46,47^. Previous studies demonstrated that the development of premature senescence was induced by p16, which is a critical regulator of cell aging and contributes to the development of interstitial fibrosis and renal tubular atrophy^4,5,48^. In this study, we found that SA-β-gal activity and α-SMA-labeled fibrosis were increased along with p16 protein levels in human pulmonary tissues.

In summary, our results demonstrate that TIME signaling mediates SAPF by promoting cell senescence and stimulating TGF-β1 and IL-11 secretion and collagen 1 production in aging pulmonary fibroblasts and the EMT in aging AT2 cells. Thus, these signals in aging fibroblasts or AT2 cells may be therapeutic targets for preventing SAPF.

### Supplementary information

Supplementary informations 1–5
