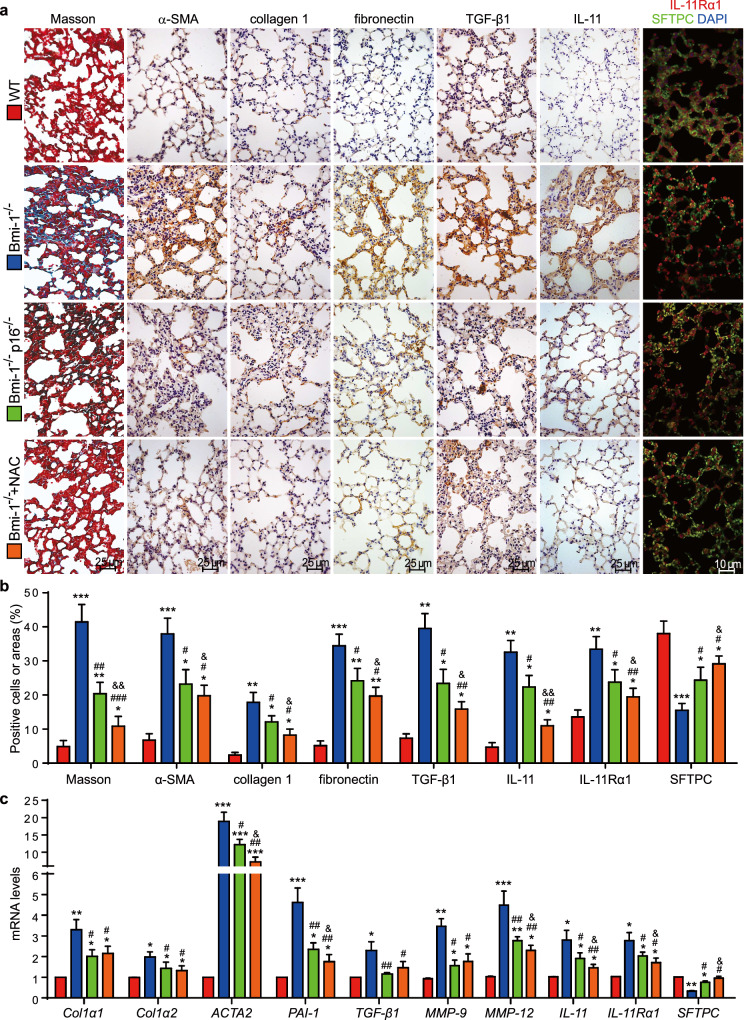# Author Correction: TGF-β1/IL-11/MEK/ERK signaling mediates senescence-associated pulmonary fibrosis in a stress-induced premature senescence model of *Bmi-1* deficiency

**DOI:** 10.1038/s12276-024-01276-1

**Published:** 2024-06-26

**Authors:** Haiyun Chen, Hongjie Chen, Jialong Liang, Xin Gu, Jiawen Zhou, Chunfeng Xie, Xianhui Lv, Rong Wang, Qing Li, Zhiyuan Mao, Haijian Sun, Guoping Zuo, Dengshun Miao, Jianliang Jin

**Affiliations:** 1 Research Center for Bone and Stem Cells, Department of Human Anatomy; Key Laboratory for Aging & Disease; The State Key Laboratory of Reproductive Medicine, Nanjing Medical University, Nanjing, Jiangsu 211166 China; 2 Antiaging Research Laboratory, Friendship Plastic Surgery Hospital, Nanjing Medical University, Nanjing, Jiangsu 210029 China; 3 Department of Nutrition and Food Safety, School of Public Health, Nanjing Medical University, Nanjing, Jiangsu 211166 China; 4 Department of Science and Technology, Jiangsu Jiankang Vocational College, Nanjing, Jiangsu 210029 China; 5 The Laboratory Center for Basic Medical Sciences, Nanjing Medical University, Nanjing, Jiangsu 211166 China

Correction to: *Experimental & Molecular Medicine* 10.1038/s12276-019-0371-7, published online 21 January 2020

In this article, the authors have just realized the wrong usage of α-SMA immunohistochemical image in the group of *Bmi-1*^*−/−*^ mice administered with NAC, using instead the α-SMA immunohistochemical image from the *Bmi-1*^*−/−*^*p16*^*−/−*^ group in Fig. 3a. The correct figure should have appeared as shown below. This is still consistent with quantitative data presented in Fig. 3b. Conclusion is not affected by the error.

The authors apologize for any inconvenience caused.

The original article has been corrected.