# Supplementary material for: TGF-β1/IL-11/MEK/ERK signaling mediates senescence-associated pulmonary fibrosis in a stress-induced premature senescence model of Bmi-1 deficiency
Source: Exp Mol Med. 2020 Jan 21;52(1):130–51. doi: 10.1038/s12276-019-0371-7 (PMC7000795; doi:10.1038/s12276-019-0371-7)
Supplement: Supplementary file 1 — Supplementary informations 1–5 [file 12276_2019_371_MOESM1_ESM.pdf]

S1: Figure S1-S5

Figure S1

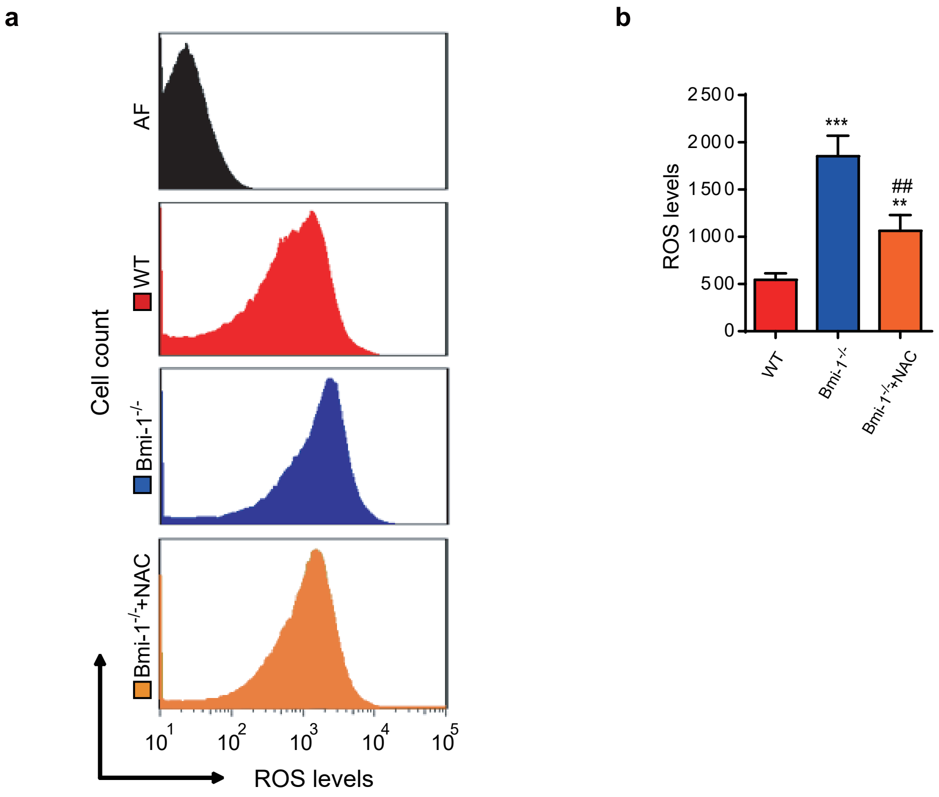

Figure S2

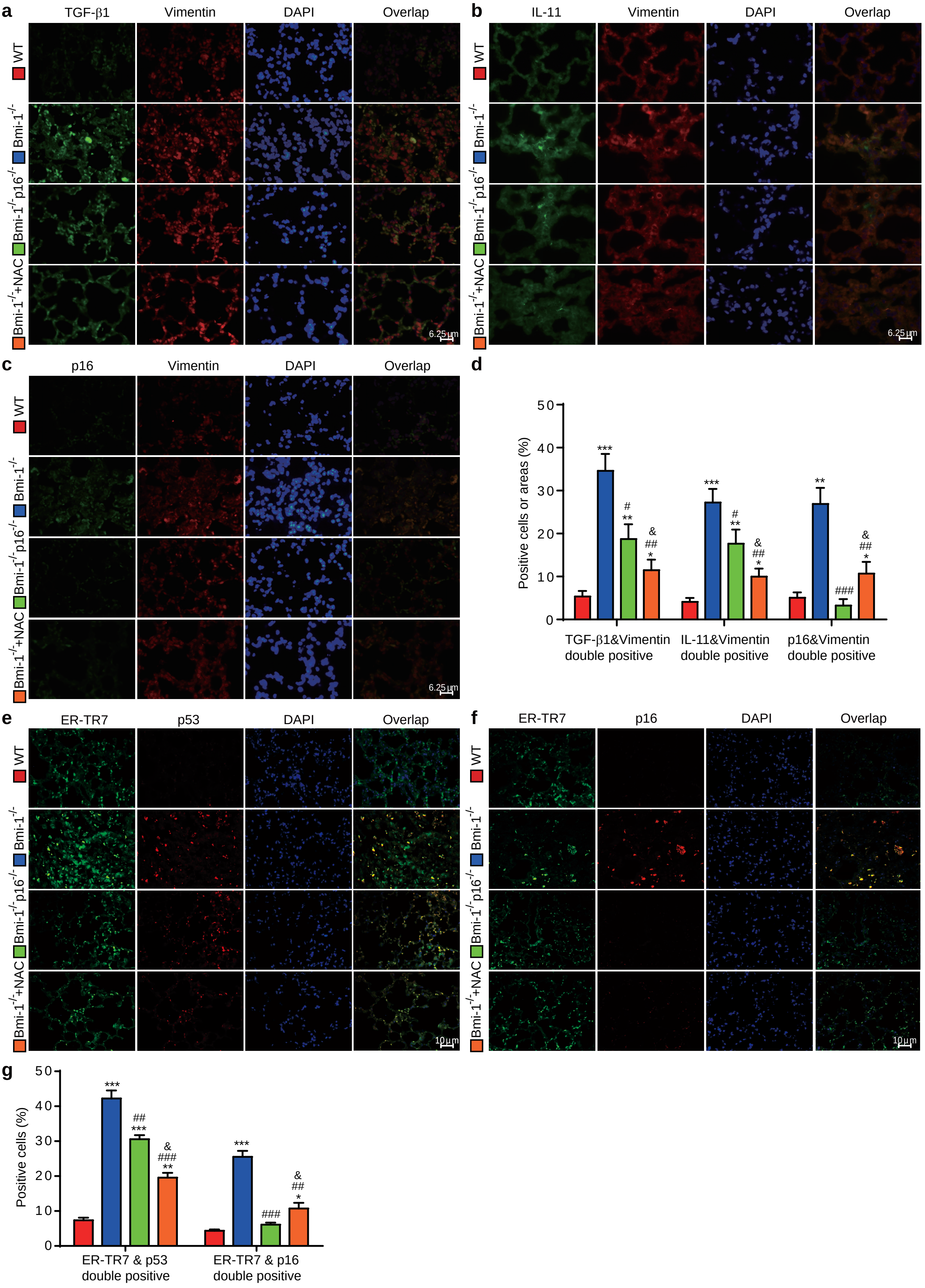

a
TGF-β1
Vimentin
DAPI
Overlap
WT
Bmi-1-/-
Bmi-1-/-p16-/-
Bmi-1-/-+NAC
6.25 μm
b
IL-11
Vimentin
DAPI
Overlap
6.25 μm
c
p16
Vimentin
DAPI
Overlap
6.25 μm
d
Positive cells or areas (%)
50
40
30
20
10
0
TGF-β1&Vimentin double positive
IL-11&Vimentin double positive
p16&Vimentin double positive
e
ER-TR7
p53
DAPI
Overlap
10 μm
f
ER-TR7
p16
DAPI
Overlap
10 μm
g
Positive cells (%)
ER-TR7 & p53 double positive
ER-TR7 & p16 double positive

Figure S3

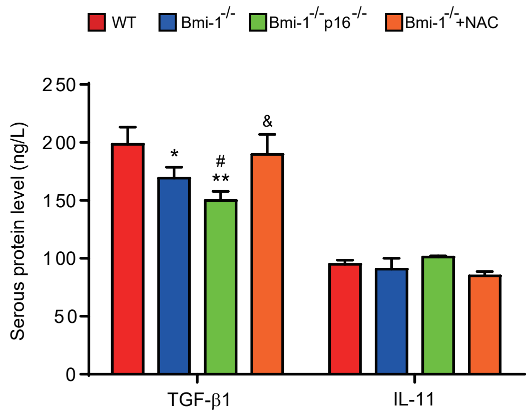

WT
Bmi-1-/-
Bmi-1-/-p16-/-
Bmi-1-/-+NAC
Serous protein level (ng/L)
250
200
150
100
50
0
*
#
**
&
TGF-β1
IL-11

Figure S4

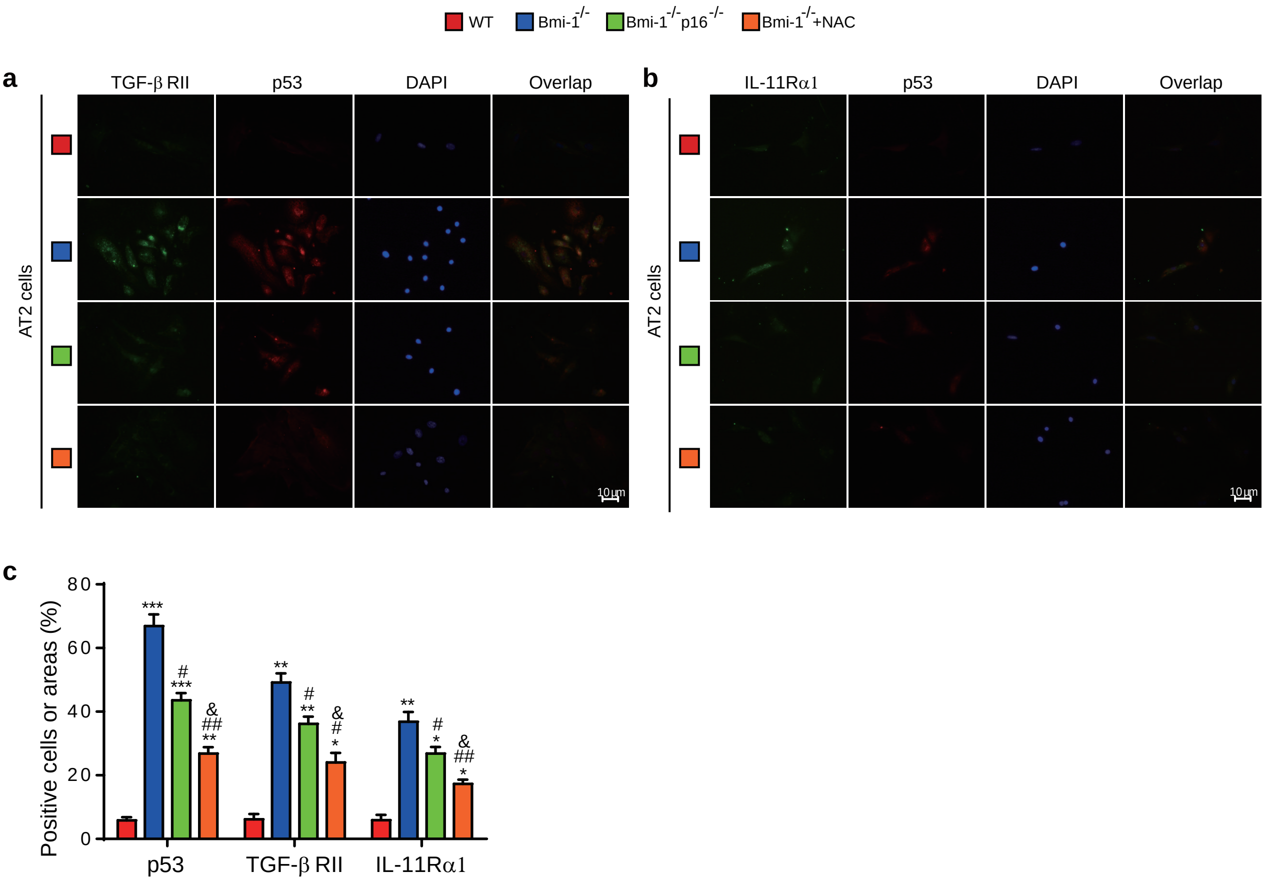

WT
Bmi-1-/-
Bmi-1-/-p16-/-
Bmi-1-/-+NAC
a
TGF-β RII
p53
DAPI
Overlap
AT2 cells
10μm
b
IL-11Rα1
p53
DAPI
Overlap
AT2 cells
10μm
c
Positive cells or areas (%)
80
60
40
20
0
***
#
***
&
##
**
**
#
**
&
#
*
**
#
*
&
##
*
p53
TGF-β RII
IL-11Rα1

Figure S5

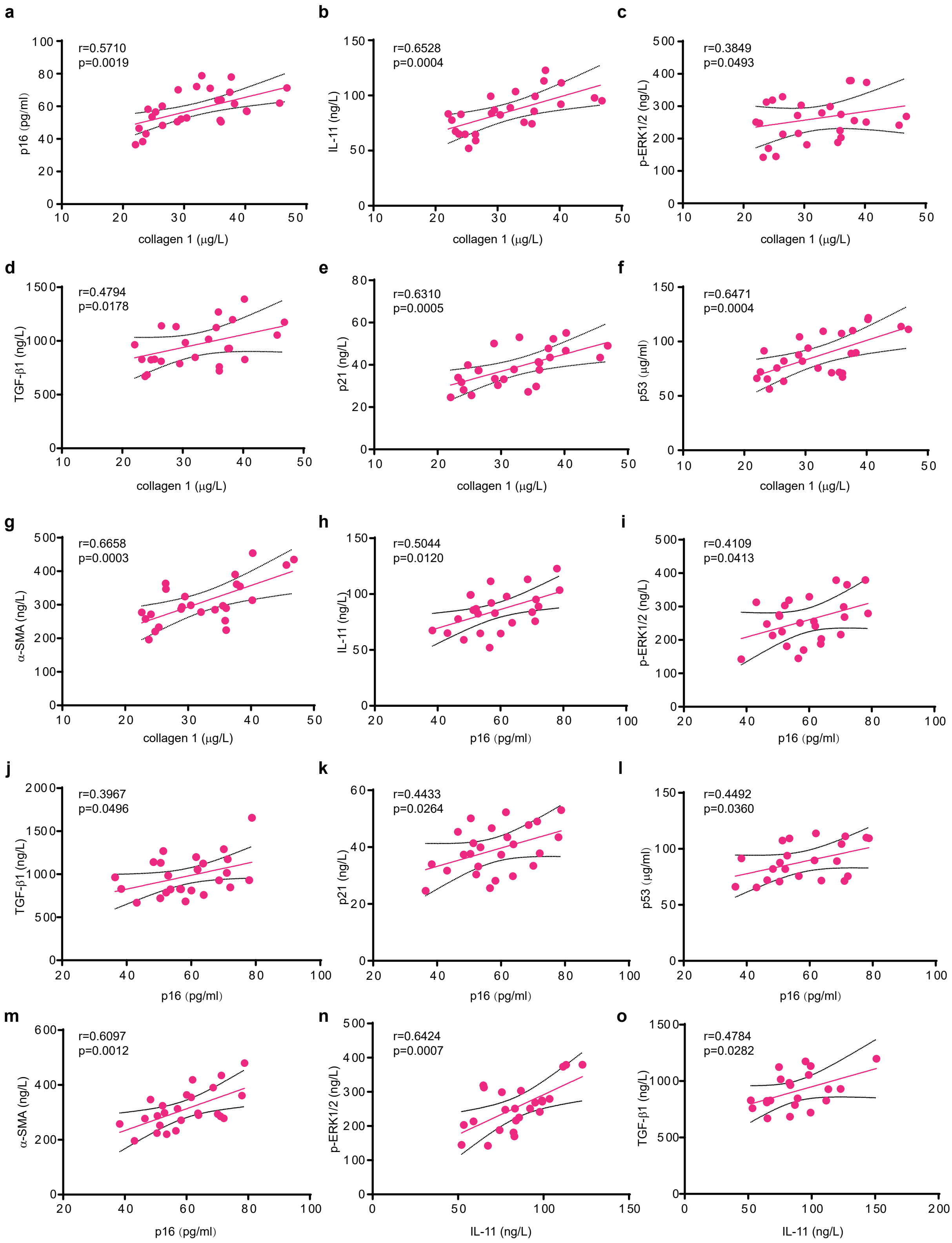

a
r=0.5710
p=0.0019
p16 (pg/ml)
collagen 1 (μg/L)
b
r=0.6528
p=0.0004
IL-11 (ng/L)
collagen 1 (μg/L)
c
r=0.3849
p=0.0493
p-ERK1/2 (ng/L)
collagen 1 (μg/L)
d
r=0.4794
p=0.0178
TGF-β1 (ng/L)
collagen 1 (μg/L)
e
r=0.6310
p=0.0005
p21 (ng/L)
collagen 1 (μg/L)
f
r=0.6471
p=0.0004
p53 (μg/ml)
collagen 1 (μg/L)
g
r=0.6658
p=0.0003
α-SMA (ng/L)
collagen 1 (μg/L)
h
r=0.5044
p=0.0120
IL-11 (ng/L)
p16 (pg/ml)
i
r=0.4109
p=0.0413
p-ERK1/2 (ng/L)
p16 (pg/ml)
j
r=0.3967
p=0.0496
TGF-β1 (ng/L)
p16 (pg/ml)
k
r=0.4433
p=0.0264
p21 (ng/L)
p16 (pg/ml)
l
r=0.4492
p=0.0360
p53 (μg/ml)
p16 (pg/ml)
m
r=0.6097
p=0.0012
α-SMA (ng/L)
p16 (pg/ml)
n
r=0.6424
p=0.0007
p-ERK1/2 (ng/L)
IL-11 (ng/L)
o
r=0.4784
p=0.0282
TGF-β1 (ng/L)
IL-11 (ng/L)

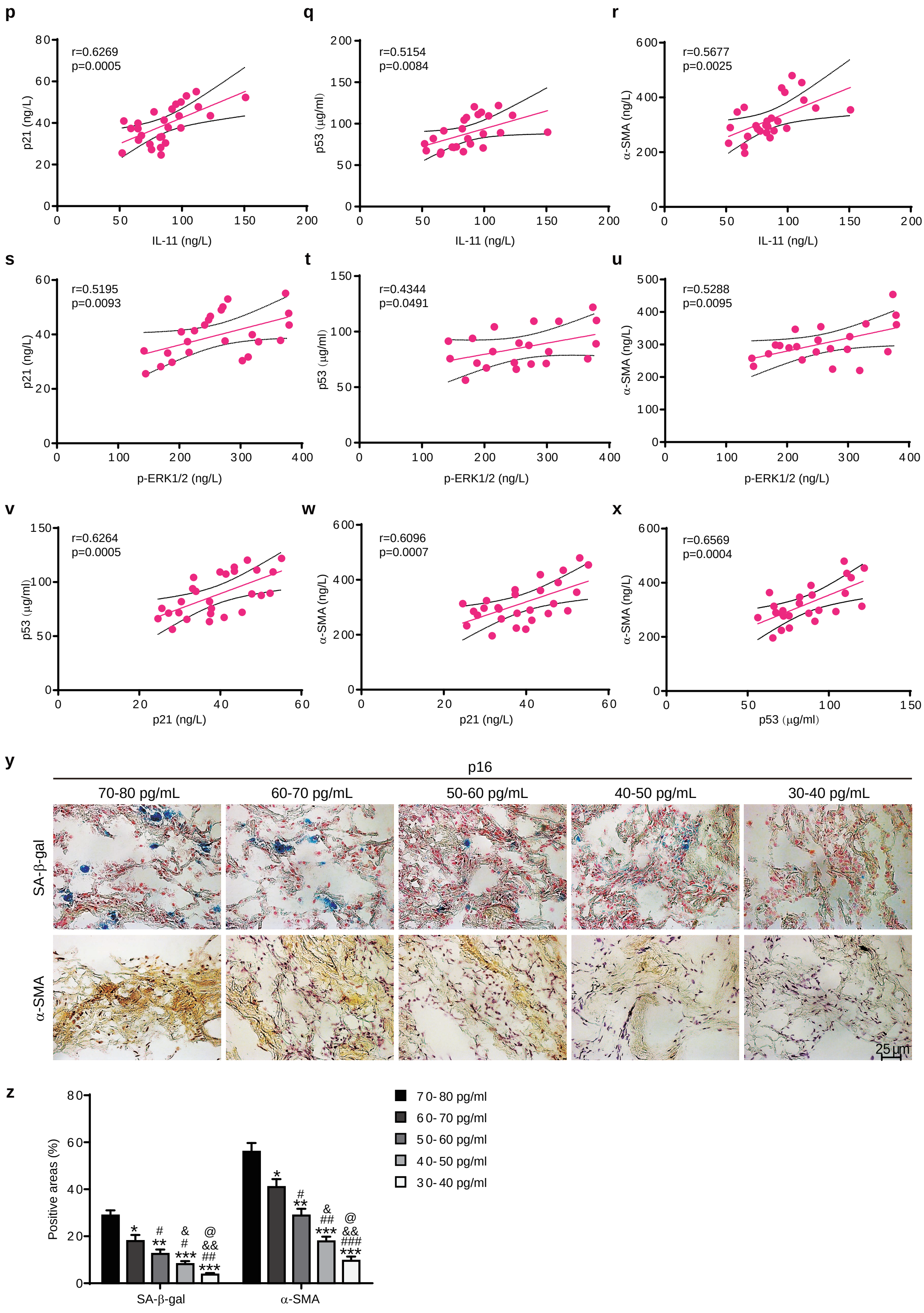
p
r=0.6269
p=0.0005
p21 (ng/L)
IL-11 (ng/L)
q
r=0.5154
p=0.0084
p53 (μg/ml)
IL-11 (ng/L)
r
r=0.5677
p=0.0025
α-SMA (ng/L)
IL-11 (ng/L)
s
r=0.5195
p=0.0093
p21 (ng/L)
p-ERK1/2 (ng/L)
t
r=0.4344
p=0.0491
p53 (μg/ml)
p-ERK1/2 (ng/L)
u
r=0.5288
p=0.0095
α-SMA (ng/L)
p-ERK1/2 (ng/L)
v
r=0.6264
p=0.0005
p53 (μg/ml)
p21 (ng/L)
w
r=0.6096
p=0.0007
α-SMA (ng/L)
p21 (ng/L)
x
r=0.6569
p=0.0004
α-SMA (ng/L)
p53 (μg/ml)
y
p16
70-80 pg/mL
60-70 pg/mL
50-60 pg/mL
40-50 pg/mL
30-40 pg/mL
SA-β-gal
α-SMA
25 μm
z
Positive areas (%)
SA-β-gal
α-SMA
70-80 pg/ml
60-70 pg/ml
50-60 pg/ml
40-50 pg/ml
30-40 pg/ml

S2: Figure S1-S5 Legends

Figure S1 NAC treatment decreased pulmonary ROS levels in *Bmi-1*$^{-/-}$ mice

(a) Pulmonary ROS from 7-week-old WT, *Bmi-1*$^{-/-}$ and NAC-treated *Bmi-1*$^{-/-}$ (*Bmi-1*$^{-/-}$+NAC) mice by flow cytometry. AF: auto fluorescence. (b) Measurements for ROS. Six biological replicates were used per experiment. Six mice per group were used for experiments. Values are means ± SEM of six determinations per group. **P < 0.01, ***P < 0.001 compared with WT group; $^{\#\#}$P < 0.01 compared with *Bmi-1*$^{-/-}$ group.

Figure S2 NAC treatment inhibited expression of TGF-β1 and IL-11 in *Bmi-1*$^{-/-}$ more than *p16* deletion pulmonary fibroblasts

Representative micrographs of paraffin-embedded pulmonary sections were immunofluorescently stained for (a) TGF-β1 and vimentin, (b) IL-11 and vimentin, or (c) p16 and vimentin, with DAPI for nuclei. (d) Percentage of double-positive areas relative to total for A-C. (e) Representative micrographs of paraffin-embedded pulmonary sections were immunofluorescently stained for fibroblast marker ER-TR7 and p53 or ER-TR7 and p16. (f) Percentage of double-positive areas relative to total for d. Six mice per group was used for experiments. Values are means ± SEM of six determinations. *P < 0.05, **P < 0.01, ***P < 0.001 compared with WT group; $^{\#}$P < 0.05, $^{\#\#}$P < 0.01, $^{\#\#\#}$P < 0.001 compared with *Bmi-1*$^{-/-}$ group; &, P < 0.05 compared with *Bmi-1*$^{-/-}$*p16*$^{-/-}$ group.

Figure S3 Serous TGF-β1 and IL-11 detected by ELISA

In 7-week-old WT, *Bmi-1*$^{-/-}$, *Bmi-1*$^{-/-}$*p16*$^{-/-}$ and NAC-treated *Bmi-1*$^{-/-}$ (*Bmi-1*$^{-/-}$+NAC) mice, serous TGF-β1 and IL-11 were detected. Six mice per group were used for experiments. Values are means ± SEM of six determinations. *P < 0.05, **P < 0.01 compared with WT group; $^{\#}$P < 0.05 compared with *Bmi-1*$^{-/-}$ group; $^{\&}$P < 0.05 compared with *Bmi-1*$^{-/-}$*p16*$^{-/-}$ group.

Figure S4 NAC treatment inhibited cell senescence and expression of TGF-β receptor-type 2 (RII) or IL-11 receptor α1 (Rα1) in *Bmi-1*$^{-/-}$ more than *Bmi-1*$^{-/-}$*p16*$^{-/-}$ AT2 cells

Pulmonary AT2 cells from 7-week-old WT, *Bmi-1*$^{-/-}$, *Bmi-1*$^{-/-}$*p16*$^{-/-}$ and NAC-treated *Bmi-1*$^{-/-}$ (*Bmi-1*$^{-/-}$+NAC) mice. (a-b) Representative micrographs of cells stained immunofluorescently for TGF-β RII or IL-11 Rα1 and p53 with DAPI for nuclei. (c) Percentage of cells positive for p53, TGF-βRII, or IL-11Rα1 or positive areas relative to total. Six biological replicates were used per experiment. Values are means ± SEM of six determinations. *P < 0.05, **P < 0.01, ***P < 0.001 compared with WT group; $^{\#}$P < 0.05, $^{\#\#}$P < 0.01 compared with *Bmi-1*$^{-/-}$ group; $^{\&}$P < 0.05 compared with *Bmi-1*$^{-/-}$*p16*$^{-/-}$ group.

Figure S5 Accumulation of collagen 1 and α-SMA in lungs accompanied by human aging mediated by TIME signals

Human pulmonary tissues (27 samples) examined for p16, collagen 1, TGF-β1, IL-11

and pERK1/2 (Thr202/Tyr204) by ELISA and analyzed for correlations. (a) Collagen 1 and p16, (b) collagen 1 and IL-11, (c) collagen 1 and p-ERK (Thr202/Tyr204), (d) collagen 1 and TGF-β1, (e) collagen 1 and p21, (f) collagen 1 and p53, (g) collagen 1 and α-SMA, (h) p16 and IL-11, (i) p16 and p-ERK (Thr202/Tyr204), (j) p16 and TGF-β1, (k) p16 and p21, (l) p16 and p53, (m) p16 and α-SMA, (n) IL-11 and p-ERK (Thr202/Tyr204), (o) IL-11 and TGF-β1, (p) IL-11 and p21, (q) IL-11 and p53, (r) IL-11 and α-SMA, (s) p-ERK (Thr202/Tyr204) and p21, (t) p-ERK (Thr202/Tyr204) and p53, (u) p-ERK (Thr202/Tyr204) and α-SMA, (v) p21 and p53, (w) p21 and α-SMA, and (x) p53 and α-SMA. Gaussian distributed data were analyzed by Pearson's r and non-Gaussian distributed data were analyzed by Spearman's r. P-values were two-sided and values less than 0.05 was considered statistically significant. (y) The frozen tissue sections of human pulmonary tissues were detected the SA-β-gal activity and a-SMA immunohistological expression according to the p16 protein levels. (z) Percentage of cells positive for SA-β-gal and α-SMA positive area relative to total area. Values are mean ± SEM from 4 determinations per group. $^{*}P < 0.05$, $^{**}P < 0.01$, $^{***}P < 0.001$ compared with 70-80 pg/ml group; $^{\#}P < 0.05$, $^{\#\#}P < 0.01$, $^{\#\#\#}P < 0.001$ compared with 60-70 pg/ml group; $^{\&}P < 0.05$, $^{\&\&}P < 0.01$ compared with 50-60 pg/ml group; $^{@}P < 0.05$ compared with 40-50 pg/ml group.

S3: Complete Materials and Methods

Mice and genotyping

Adult *Bmi-1* heterozygote (*Bmi-1*$^{+/-}$) mice (129Ola/FVB/N hybrid background) were mated to generate *Bmi-1* homozygote (*Bmi-1*$^{-/-}$) and their wild-type (WT) littermates genotyped by PCR, as described previously [1-5]. *P16*$^{+/-}$ mice of the FVB N2 background were crossed to *Bmi-1*$^{+/-}$ mice to generate double-knockout (*Bmi-1*$^{-/-}$*p16*$^{-/-}$) mice and genotyped by PCR, as described previously [1,2]. This study was carried out in strict accordance with the guidelines of the Institute for Laboratory Animal Research of Nanjing Medical University in Nanjing of China. The protocol was approved by the Committee on the Ethics of Animal Experiments of Nanjing Medical University (Permit Number: IACUC-1706001).

Samples of human pulmonary tissues

Human pulmonary samples were obtained from 27 autopsies at the Department of Human Anatomy in Nanjing Medical University. Anatomical methods and all experimental protocols were approved by the Committee on the Ethics of Nanjing Medical University (Permit Number: 2019-902). Body donors, from 39- to 94-year-old, had no tumors, acquired immune deficiency syndrome, autoimmune disease, respiratory chronic infections or inflammatory disease, before they died. These human pulmonary samples were detected for ELISA assays, SA-β-gal staining and a-SMA immunohistochemical staining as follows.

Cell cultures

Pulmonary fibroblasts

Mice that were 7-weeks-old were anesthetized with 3% pentobarbital sodium (40 mg kg^{-1}) and perfused with 100 ml phosphate buffered saline (PBS) (0.01 mM PO_4^{3-}, pH 7.4). Lungs were separated with blunt dissection and rinsed three times in PBS containing 200 U/ml penicillin and 200 μg/ml streptomycin (Gibco, Grand Island, NY, USA). Then, lungs were minced and digested for 1 hour in digestive solution including 1 mg/ml collagenase D (0.30U mg^{-1} lyo., Roche Diagnostics GmbH, Mannheim, BW, Germany) and 2% FBS (v/v) (Gibco) dissolved in normal culture medium of Dulbecco's Modified Eagle Medium (DMEM)/F12 (Gibco) at 175 rpm in a constant temperature shaker at 37°C and centrifuged with 1500 rpm for 5 min [2]. Supernatants were discarded and pellets were washed with PBS for 3 times and centrifuged repeatedly. These pellets, pulmonary fibroblasts, were re-suspended in 10 ml normal culture medium of DMEM/F12 containing 10% (v/v) FBS, 100 U/ml penicillin and 100 μg/ml streptomycin and placed in 10-cm petri dishes and kept in a humidified 5% CO_2 incubator at 37 °C [2]. Half the medium was changed every 3 days. Pulmonary fibroblasts were not recovered for expansion using 0.25% trypsin-0.02% EDTA (Gibco) until 90% confluence and detected by immunofluorescence for the mesenchymal cell marker vimentin [6] (Fig. 4e). According to the Hayflick limitation [7], pulmonary fibroblasts were repeatedly passaged to purify and observed senescence phenotype. Since the third-passage pulmonary fibroblasts remarkably displayed

senescence phenotype from *Bmi-1*$^{-/-}$ mice, the third-passage pulmonary fibroblasts from WT, *Bmi-1*$^{-/-}$, *Bmi-1*$^{-/-}$*p16*$^{-/-}$ or NAC-treated *Bmi-1*$^{-/-}$ group were used in the experiments.

Alveolar type II epithelial (AT2) cells

Mice that were 7-weeks-old were anesthetized with 3% pentobarbital sodium (40 mg/kg) and perfused with 100 ml PBS. Preheated digestive solution I [(50 U/ml Dispase (Roche Diagnostics GmbH)] of 37 °C was injected into lung through the gap of tracheal cartilage rings. Then the trachea was ligated at once below the injection site to prevent leakage of digestive solution I. After cutting the trachea above the ligation point, the tracheal, main bronchus and lungs were separated with blunt dissection and immersed into preheated digestive solution II (1 ml 50 U/ml Dispase, 1 ml 1mg/ml collagenase D, and 5μl DNAase I) (Roche Diagnostics GmbH) at 120 rpm in a constant temperature shaker at 37°C for 10 min. Lungs were separated, minced and immersed into digestive solution III (0.25% trypsin-0.02% EDTA) at 120 rpm in a constant temperature shaker at 37°C for 10 min. The digestive production was sequentially filtered with a sieve of 70 μm and 40 μm and centrifuged with 1000 rpm for 5 min. Supernatants were discarded and pellets were resuspended and incubated for 45 min in 10 ml normal culture medium of DMEM/F12 containing 10% (v/v) FBS, 100 U/ml penicillin and 100 μg/ml streptomycin. The supernatants were centrifuged with 1000 rpm for 5 min. Then pellets were resuspended and incubated at 37°C for 1 h in petri dish coated with 0.5 mg/ml IgG (dissolved in Tris-base with PH 9.4) with

normal culture medium. The non-adherent cells were collected and centrifuged with 1000 rpm for 8 min [8,9]. These pellets, AT2 cells, were re-suspended in normal culture medium and kept in a humidified 5% CO_2 incubator at 37 °C. Half the medium was changed every 3 days. AT2 cells were not recovered for expansion using 0.25% trypsin-0.02% EDTA (Gibco) until 80% confluence and detected by western blot and immunofluorescence for the surfactant protein C (SFTPC) marker (Fig. 6 d, f, h and j) [10-12]. Since the second-passage AT2 cells remarkably displayed senescence phenotype from *Bmi-1*$^{-/-}$ mice, the second-passage AT2 cells from WT, *Bmi-1*$^{-/-}$, *Bmi-1*$^{-/-}$*p16*$^{-/-}$ or NAC-treated *Bmi-1*$^{-/-}$ group were used in the experiments.

Administration of drugs or reagents

N-acetylcysteine

For *in vivo* administration of N-acetylcysteine (NAC), we randomized 3-week-old *Bmi-1*$^{-/-}$ mice to water containing NAC at 1 mg/ml or ordinary drinking water. Moreover, because *Bmi-1*$^{-/-}$ mice are frail, the food of mice was soaked in NAC-treated or NAC-untreated drinking water and placed inside the cage as previously described [1,13].

For *in vitro* treatment of NAC, cells were incubated without or with NAC at 1 mM (0.163 mg/ml) [1,14]. Pulmonary fibroblasts or AT2 cells of the NAC-treated *Bmi-1*$^{-/-}$ group were isolated from 7-week-old NAC-treated *Bmi-1*$^{-/-}$ mice and continuously cultured with NAC treatment.

Exogenous recombinant mouse TGF-β1 and IL-11, MEK inhibitor and anti-IL-11

antibody

Pulmonary fibroblasts or AT2 cells were treated with TGF-β1 (5 ng/ml 48h) (Novoprotein Scientific Inc., Shanghai, China), TGF-β1 (5 ng/ml 48h) and anti-IL-11 (2 μg/ml 48h) (sc-133063, Santa Cruz Biotechnology Inc., Dallas, TX, USA), IL-11 (5 ng/ml 48h) (Novoprotein Scientific Inc.), or IL-11 (5 ng/ml 48h) and MEK inhibitor (PD98059) (10 μM 48h) (#9900, Cell Signaling Technology, Beverly, MA, USA) as previously described [15].

MG132

MG132, a proteasome inhibitor, was used to inhibit the proteasome in *Bmi-1*$^{-/-}$ Pulmonary fibroblasts (5 μM for 48h) (#474787, Sigma-Aldrich, St. Louis, MO, USA) as previously described [16].

Cell proliferation

Cell proliferation was analyzed using Cell Counting Kit-8 (CCK-8) assay kits (#C0038, Beyotime Institute of Biotechnology, Shanghai, China) as previously described [2]. Briefly, third-passage Pulmonary fibroblasts or second-passage AT2 were seeded in 96-well plates at 2000 cells well^{-1} and incubated for 0, 24, 48, 72 or 96 hours. CCK-8 of 10 μl was added to each well (100 μl DMEM/F12) and incubated with cells at 37 °C for 1 hour. Then, cells were detected by spectrophotometry at 450 nm absorbance following manufacturer's instructions and population doublings were calculated as previous described [2,4].

Conditioned medium collection

Third-passage pulmonary fibroblasts were cultured in DMEM/F12 (without phenol red; Gibco) without FBS for 24 hours. Supernatants were collected and filtered with MILLEX-GP 0.22-μm filters (Merck Millipore Ltd. Co., Cork, Munster, Ireland) to remove cell debris and concentrated to 10% volume with Amicon Ultra-4 centrifugal ultrafiltration tubes (NMWL 3KDa) (Merck Millipore Ltd. Co.) for use as conditioned medium as previously described [2].

Enzyme-linked immunosorbent assay

Assays were according to the manufacturer's instructions and assessed by densitometry analysis as previously described [17]. ELISA kits (Yifeixue Biotechnology, Nanjing, China) were used to detect concentrations of mouse-derived TGF-β1 (#M00081) and IL-11 (#M00807) in CM and serum of mice; and human-derived TGF-β1 (#H00080), IL-11 (#H00439), p16 (#H01273), collagen 1 (#H00179), pERK1/2(Thr202/Tyr204) (#H01101), p53 (#H00199), p21 (#H00200) and α-SMA (#H01041) in human pulmonary tissue.

Intracellular ROS analysis

Total pulmonary cells from 7-week-old mice were incubated with 5 mM 2', 7'-dichlorofluorescin diacetate (DCFDA) (Invitrogen Inc., Carlsbad, CA, USA) and placed in a shaker without light at 37°C for 30 min, washed 3 times and followed immediately by flow cytometry analysis in a FACScalibur flow cytometer (Becton

Dickinson, Heidelberg, Germany) [1,13].

Pulmonary function analysis

Mice that were 7-weeks-old were anesthetized with 3% pentobarbital sodium (40 mg/kg), underwent tracheostomies and mechanically ventilated at an initial baseline challenge using the FinePointe RC system (Buxco Research Systems, Wilmington, NC, USA) to directly evaluate lung ventilatory resistance and compliance, including peak inspiratory flow, frequency, tidal volume, lung resistance, dynamic compliance, minute volume, static compliance, and elastance [18-20].

Preparation of pulmonary sections

Mice were anesthetized with 3% pentobarbital sodium (40 mg/kg) at 7 weeks of age and perfused with 100 ml PBS and periodate-lysine-paraformaldehyde (PLP) solution (4% paraformaldehyde containing 0.075 M lysine and 0.01 M sodium periodate) in turn. Pulmonary samples were cut into small pieces and post fixed in PLP solution overnight at 4°C. For histochemistry or immunohistochemistry, sections were dehydrated in a series of graded ethanol solutions, embedded in paraffin and cut into 5-μm sections using a rotary microtome (Leica Biosystems Nussloch GmbH, Nussloch, Germany) as previously described [1,2].

Human frozen pulmonary samples were cut on a freezing microtome (Thermo Scientific Cryotome FSE Cryostats, Loughborough, Leicestershire) at 7 μm thickness for SA-β-gal staining and a-SMA immunohistochemical staining.

Histology staining

For histochemical or immunohistochemical staining, serial paraffin sections were deparaffinized and rehydrated.

Pre-embedding SA-β-gal staining

Pulmonary samples were cut into small pieces and washed three times for 30 min in LacZ wash buffer (2 mM $MgCl_2$, 0.01% sodium deoxycholate, 0.02% nonidet-P40 in PBS, pH 6.0) following fixing for 2 hours in PLP. Staining was conducted in the solution (0.5 mg/ml X-gal, 5 mM potassium ferrocyanide and 5 mM potassium ferricyanide (Sigma-Aldrich) dissolved in LacZ wash buffer) in a constant temperature shaker at 37°C overnight with protection from light as previously described [1,2,21]. Then, these samples were re-fixed in PLP overnight, dehydrated in a series of graded ethanol solutions, embedded in paraffin and cut into 5-μm sections using a rotary microtome as previously described [1].

SA-β-gal staining

Frozen sections of human pulmonary samples were stained with Senescence β-Galactosidase Staining Kit (#C0602, Beyotime Institute of Biotechnology, Shanghai, China) according to the manufacturer's instructions following previously described methods [22]. Briefly, frozen sections were fixed in the fixative at room temperature for 15 min. These sections were rinsed with PBS for 3 times and then incubated with the fresh SA-β-gal staining working solution overnight at 37°C [22].

Masson's trichrome staining

Serial paraffin sections were generated with Masson detection kits (#D026, Nanjing Jiancheng Bioengineering Institute, Nanjing, Jiangsu, China) according to the manufacturer's instructions as previously described methods [1,2].

Immunohistochemical staining

Serial paraffin sections were generated for antigen retrieval, steamed for 20 minutes in Sodium Citrate Buffer (10 mM sodium citrate acid, 0.05% Tween-20, PH 6.0) followed by blocking of endogenous peroxidase (3% H_2O_2) and pre-incubation with serum as previously described [1-3]. Primary antibodies were against p53 (#2524, Cell Signaling Technology, Beverly, MA, USA), 8-OHdG (ab62623, Abcam, Cambridge, MA, USA), IL-1β (ab9722, Abcam), IL-6 (sc-1265, Santa Cruz Biotechnology Inc.), TNF-α (sc-52746, Santa Cruz Biotechnology Inc.), α-SMA (ab28052, Abcam), collagen 1 (#1310-08, Southern Biotech, Birmingham, AL, USA), fibronectin (#SAB4500974, Sigma-Aldrich), TGF-β1 (ab64715, Abcam), and IL-11 (sc-133063, Santa Cruz Biotechnology Inc.). After washing, sections were incubated with secondary antibody (biotinylated IgG; Sigma-Aldrich), washed and processed using Vectastain ABC-HRP kits (Vector Laboratories Inc., Burlingame, CA, USA). Sections were counterstained with hematoxylin and mounted with Biomount medium [1,2].

Immunofluorescent staining of pulmonary sections

Serial paraffin sections were generated for antigen retrieval and pre-incubation with serum as the immunohistochemical staining. Primary antibodies against CD3

(sc-20047, Santa Cruz Biotechnology Inc.), F4/80 (sc-377009, Santa Cruz Biotechnology Inc.), IL-11Rα1 (sc-130920, Santa Cruz Biotechnology Inc.), SFTPC (ab211326, Abcam), TGF-β1 (ab64715, Abcam), IL-11 (sc-133063, Santa Cruz Biotechnology Inc.), vimentin (#5741, Cell Signaling Technology), p53 (#2524, Cell Signaling Technology), p16 (#MA5-17142, Invitrogen Inc.), Fibroblast Marker (ER-TR7) (sc-73355, Santa Cruz Biotechnology Inc.) and TGF-β RII (sc-17792, Santa Cruz Biotechnology Inc.), and affinity-purified Alexa Fluor 488-conjugated secondary antibody and 594-conjugated secondary antibody (Life Technologies Corporation, USA) were used. Nuclei were labeled with DAPI (Sigma-Aldrich, USA) and mounted with medium to prevent quenching (Vector Laboratories Inc., USA) as previously described [2].

Cytology staining

Cells seeded on Lab-Tek®II Chamber Slide™ system (Thermo Fisher Scientific Inc., Rochester, NY, USA) were fixed with PLP solution for 1 hour [2,23].

SA-β-gal staining

SA-β-gal staining of cells also used Senescence β-Galactosidase Staining Kit (#C0602, Beyotime Institute of Biotechnology) according to the manufacturer's instructions as previously described [22]. Briefly, the medium of cell culture was discarded. Cells were rinsed with PBS for 3 times, fixed with 1 ml of fixative solution for 30 min, washed with PBS for 3 times again, and reacted with the with the fresh SA-β-gal staining working solution overnight at 37°C [22].

Immunofluorescent staining of cells

Cells were pre-incubated with serum. Primary antibodies against IL-11 (sc-133063, Santa Cruz Biotechnology Inc.), vimentin (#5741, Cell Signaling Technology), p53 (#2524, Cell Signaling Technology), TGFβ RII (sc-17792, Santa Cruz Biotechnology Inc.), IL-11Rα1 (sc-130920, Santa Cruz Biotechnology Inc.), α-SMA (ab28052, Abcam), SFTPC (ab211326, Abcam), p16 (#MA5-17142, Invitrogen Inc.), ERK1/2 (#4695, Cell Signaling Technology), and pERK1/2(Thr202/Tyr204) (#4370, Cell Signaling Technology) and affinity-purified Alexa Fluor 488-conjugated secondary antibody and 594-conjugated secondary antibody (Life Technologies Corporation, USA) were used. Nuclei were labeled with DAPI (Sigma-Aldrich, USA) and mounted with medium to prevent quenching (Vector Laboratories Inc., USA) [2,23].

RNA extraction and real-time RT-PCR

RNA was extracted from lungs of 7-week-old mice using TRIzol reagent (#15596, Invitrogen Inc.) according to the manufacturer's protocol. Levels of mRNA in pulmonary samples were quantified by real-time RT-PCR as previously described [1,2]. Real-time RT-PCR primers are in Table S1.

Western Blots

Lungs from 7-week-old mice were dissected. Nuclear and cytoplasm fractions of pulmonary fibroblasts were extracted using NE-PER™ Nuclear and Cytoplasmic

Extraction Reagents (Pierce Biotechnology, Rockford, IL, USA) according to the manufacturer's protocol [23]. Pulmonary samples, pulmonary fibroblasts, AT2 cells, and the nuclear and cytoplasm fractions of pulmonary fibroblasts were immediately placed into RIPA lysis buffer (#P0013B, Beyotime Institute of Biotechnology) containing a cocktail of proteinase inhibitors and phosphatase inhibitors (#4906845001, Roche Diagnostics Corp., Basel, Switzerland) and phenylmethanesulfonyl fluoride (PMSF) (#ST506, Beyotime Institute of Biotechnology) for protein extraction. Western blots were performed as previously described [1,2]. Primary antibodies were against p16 (ab211542, Abcam), p19 (sc-1665, Santa Cruz Biotechnology Inc.), p53 (sc-126, Santa Cruz Biotechnology Inc.), p21 (sc-471, Santa Cruz Biotechnology Inc.), 8-OHdG (ab62623, Abcam), SFTPC (ab211326, Abcam), collagen 1 (#1310-08, Southern Biotech), α-SMA (ab28052, Abcam), TGF-β1 (ab64715, Abcam), TGF-β RII (sc-17792, Santa Cruz Biotechnology Inc.), Smad2 (sc-101153, Santa Cruz Biotechnology Inc.), phospho-Smad2 (Ser465/467) (#3108, Cell Signaling Technology), phospho-Smad2/3 (Ser423/425) (sc-11769, Santa Cruz Biotechnology Inc.), IL-11 (sc-133063, Santa Cruz Biotechnology Inc.), IL-11Rα1 (sc-130920, Santa Cruz Biotechnology Inc.), MEK1/2 (sc-81504, Santa Cruz Biotechnology Inc.), phospho-MEK1/2 (sc-81503, Santa Cruz Biotechnology Inc.), ERK1/2 (#4695, Cell Signaling Technology), pERK1/2(Thr202/Tyr204) (#4370, Cell Signaling Technology), eIF4E (sc-9976, Santa Cruz Biotechnology Inc.), p-eIF4E (#9741, Cell Signaling Technology), RSK (sc-74575, Santa Cruz Biotechnology Inc.), p-RSK(Ser380) (sc-377526, Santa Cruz

Biotechnology Inc.), or Snail (#3879, Cell Signaling Technology). Histone H3 (#4499, Cell Signaling Technology) was the loading control for the nuclear fraction and β-actin (BS6007M, Bioworld Technology, St. Louis Park, MN, USA) or Gapdh (AP0063, Bioworld Technology) for the cytoplasm fraction and total cell protein. After incubating with HRP-conjugated secondary antibody (Sigma, USA) for 1 h, immunoreactive bands were visualized by treatment of enhanced chemiluminescence reagent and exposure to hyperfilm-ECL detection kit (Amersham Pharmacia, New Jersey, USA). Band intensity was measured using Image J version 1.29 (National Institutes of Health) as previously described [1].

Co-immunoprecipitation

This analysis was performed with Pierce® Co-Immunoprecipitation (Co-IP) Kits (#26149, Pierce Biotechnology, Rockford, IL, USA) as previously described [24]. Pulmonary fibroblasts from WT mice were treated with TGF-β1 and cytoplasm proteins extracted for p16 (ab211542, Abcam), Bmi-1 (#6964, Cell Signaling Technology), ERK1/2 (#4695, Cell Signaling Technology) or pERK1/2(Thr202/Tyr204) (#4370, Cell Signaling Technology) co-immunoprecipitation analysis. Co-precipitates or total cytoplasm lysates were detected with ERK1/2 (#4695, Cell Signaling Technology) and pERK1/2(Thr202/Tyr204) (#4370, Cell Signaling Technology) or p16 (ab211542, Abcam) for Western blots which was the same as the above methods.

Duolink Proximity Ligation Assay (PLA)

Duolink PLA in situ-fluorescence (Sigma-Aldrich) was performed as the manufacturer's instructions with Duolink in situ PLA probe anti-mouse PLUS (#DUO92001), Duolink in situ PLA probe anti-rabbit MINUS (#DUO92005), Duolink in situ detection reagents Red (#DUO92008) and Duolink in situ wash buffers-fluorescence (#DUO82049). Pulmonary fibroblasts from *Bmi-1*$^{-/-}$ mice were treated with TGF-β1 and detected for p16 (MA5-17142, Thermo Fisher Scientific, IL, USA) & ERK1/2 (#4695, Cell Signaling Technology), and p16 (MA5-17142, Thermo Fisher Scientific, IL, USA) & pERK1/2(Thr202/Tyr204) (#4370, Cell Signaling Technology). PLA signal (λ-excitation 594 nm, λ-emission 624 nm; Texas Red) was analyzed as previously described [25]. Nuclei were labeled with DAPI (Sigma-Aldrich, USA) and mounted with medium to prevent quenching (Vector Laboratories Inc., USA).

Statistical analysis

All analyses were performed using GraphPad Prism software (Version 6.07; GraphPad Software Inc., San Diego, CA, USA) as previously described [15]. Briefly, Measurement data were described as mean ± SEM fold-change over vehicle group and analyzed by Student's *t*-test and one-way ANOVA to compare differences among groups. Qualitative data was described as percentages and analyzed using chi-square tests as indicated. P-values were two-sided and less than 0.05 was considered statistically significant [1,2]. Correlation of Gaussian distributed data were analyzed by

Pearson's r and non-Gaussian distributed data were analyzed by Spearman's r. P-values were two-sided and values less than 0.05 was considered statistically significant.

References

1. Jin, J., *et al.* Bmi-1 plays a critical role in protection from renal tubulointerstitial injury by maintaining redox balance. *Aging Cell* **13**, 797-809 (2014).
2. Jin, J., *et al.* P16 (INK4a) Deletion Ameliorated Renal Tubulointerstitial Injury in a Stress-induced Premature Senescence Model of Bmi-1 Deficiency. *Sci Rep* **7**, 7502 (2017).
3. Xie, C., *et al.* Anti-aging Effect of Transplanted Amniotic Membrane Mesenchymal Stem Cells in a Premature Aging Model of Bmi-1 Deficiency. *Sci Rep* **5**, 13975 (2015).
4. Zhang, H.W., *et al.* Defects in mesenchymal stem cell self-renewal and cell fate determination lead to an osteopenic phenotype in Bmi-1 null mice. *J Bone Miner Res* **25**, 640-652 (2010).
5. Lv, X., *et al.* Bmi-1 plays a critical role in the protection from acute tubular necrosis by mobilizing renal stem/progenitor cells. *Biochem Biophys Res Commun* **482**, 742-749 (2017).
6. Cutz, E., Chami, R., Dell, S., Langer, J. & Manson, D. Pulmonary interstitial glycogenosis associated with a spectrum of neonatal pulmonary disorders. *Hum Pathol* **68**, 154-165 (2017).
7. Hayflick, L. Recent advances in the cell biology of aging. *Mechanisms of ageing and development* **14**, 59-79 (1980).
8. Gadepalli, V.S., Vaughan, C. & Rao, R.R. Isolation and characterization of murine multipotent lung stem cells. *Methods Mol Biol* **962**, 183-191 (2013).
9. Jiang, C., *et al.* Serpine 1 induces alveolar type II cell senescence through activating p53-p21-Rb pathway in fibrotic lung disease. *Aging Cell* **16**, 1114-1124 (2017).
10. Xu, X., *et al.* Evidence for type II cells as cells of origin of K-Ras-induced distal lung adenocarcinoma. *Proc Natl Acad Sci U S A* **109**, 4910-4915 (2012).
11. Yamamoto, Y., *et al.* Long-term expansion of alveolar stem cells derived from human iPS cells in organoids. *Nat Methods* **14**, 1097-1106 (2017).
12. Lu, J., *et al.* Baicalin alleviates radiation-induced epithelial-mesenchymal transition of primary type II alveolar epithelial cells via TGF-beta and ERK/GSK3beta signaling pathways. *Biomed Pharmacother* **95**, 1219-1224 (2017).
13. Liu, J., *et al.* Bmi1 regulates mitochondrial function and the DNA damage response pathway. *Nature* **459**, 387-392 (2009).
14. Ambrogini, E., *et al.* FoxO-mediated defense against oxidative stress in osteoblasts is indispensable for skeletal homeostasis in mice. *Cell Metab* **11**, 136-146 (2010).
15. Schafer, S., *et al.* IL-11 is a crucial determinant of cardiovascular fibrosis. *Nature* **552**, 110-115 (2017).
16. Harhouri, K., *et al.* MG132-induced progerin clearance is mediated by autophagy activation and splicing regulation. *EMBO Mol Med* **9**, 1294-1313 (2017).
17. Wang, W., *et al.* Typhonium giganteum Lectin Exerts A Pro-Inflammatory Effect on RAW

264.7 via ROS and The NF-kappaB Signaling Pathway. *Toxins (Basel)* **9**(2017).

18. Li, Y.*, et al.* Oligomeric proanthocyanidins attenuate airway inflammation in asthma by inhibiting dendritic cells maturation. *Mol Immunol* **91**, 209-217 (2017).
19. Zhou, Y.*, et al.* Aucubin Alleviates Bleomycin-Induced Pulmonary Fibrosis in a Mouse Model. *Inflammation* **40**, 2062-2073 (2017).
20. Hashimoto, M.*, et al.* Elimination of p19(ARF)-expressing cells enhances pulmonary function in mice. *JCI Insight* **1**, e87732 (2016).
21. Jin, J.*, et al.* An improved transplantation strategy for mouse mesenchymal stem cells in an acute myocardial infarction model. *PLoS One* **6**, e21005 (2011).
22. Chen, B.*, et al.* Downregulation of ribosomal protein S6 inhibits the growth of non-small cell lung cancer by inducing cell cycle arrest, rather than apoptosis. *Cancer letters* **354**, 378-389 (2014).
23. Gu, X.*, et al.* 1, 25-dihydroxy-vitamin D3 with tumor necrosis factor-alpha protects against rheumatoid arthritis by promoting p53 acetylation-mediated apoptosis via Sirt1 in synoviocytes. *Cell Death Dis* **7**, e2423 (2016).
24. Liu, X.*, et al.* Androgen receptor and heat shock protein 27 co-regulate the malignant potential of molecular apocrine breast cancer. *J Exp Clin Cancer Res* **37**, 90 (2018).
25. Brueggemann, L.I.*, et al.* Differential protein kinase C-dependent modulation of Kv7.4 and Kv7.5 subunits of vascular Kv7 channels. *The Journal of biological chemistry* **289**, 2099-2111 (2014).

S4: Alignment of every two proteins

1. MAPK1 & MAPK8

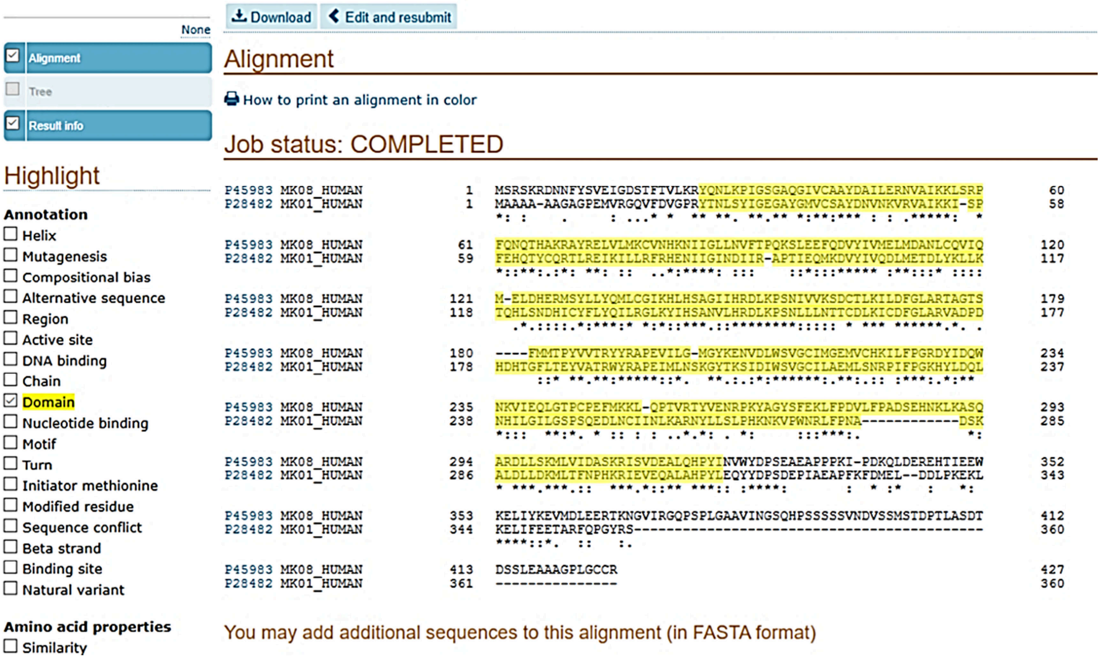

2. MAPK3 & MAPK8

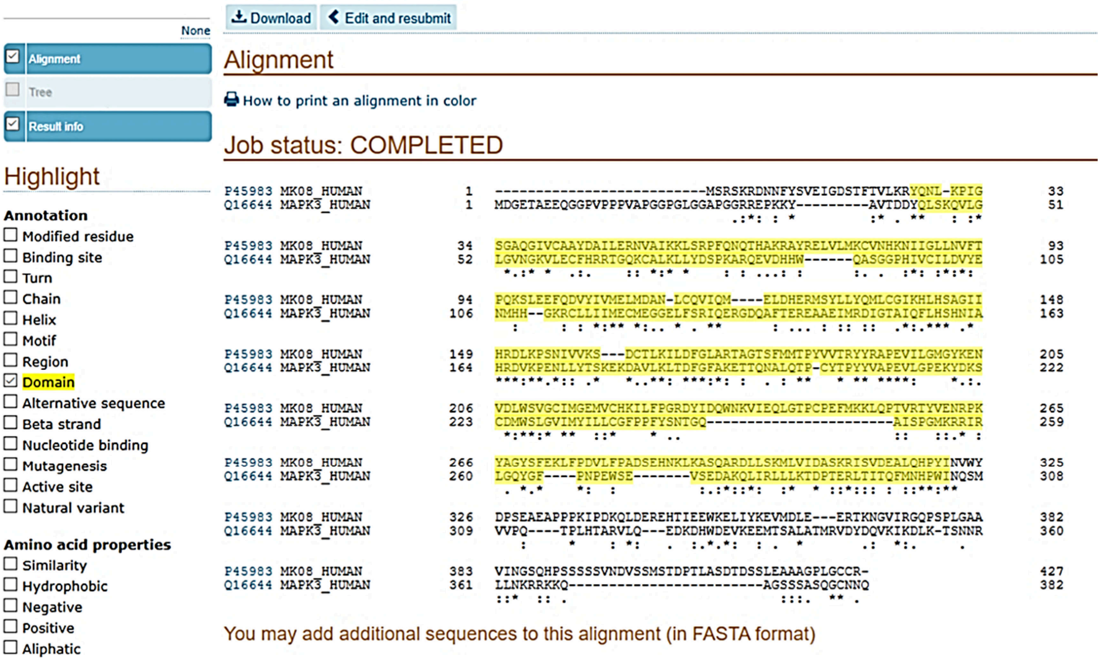

3. MAPK1 & MAPK10

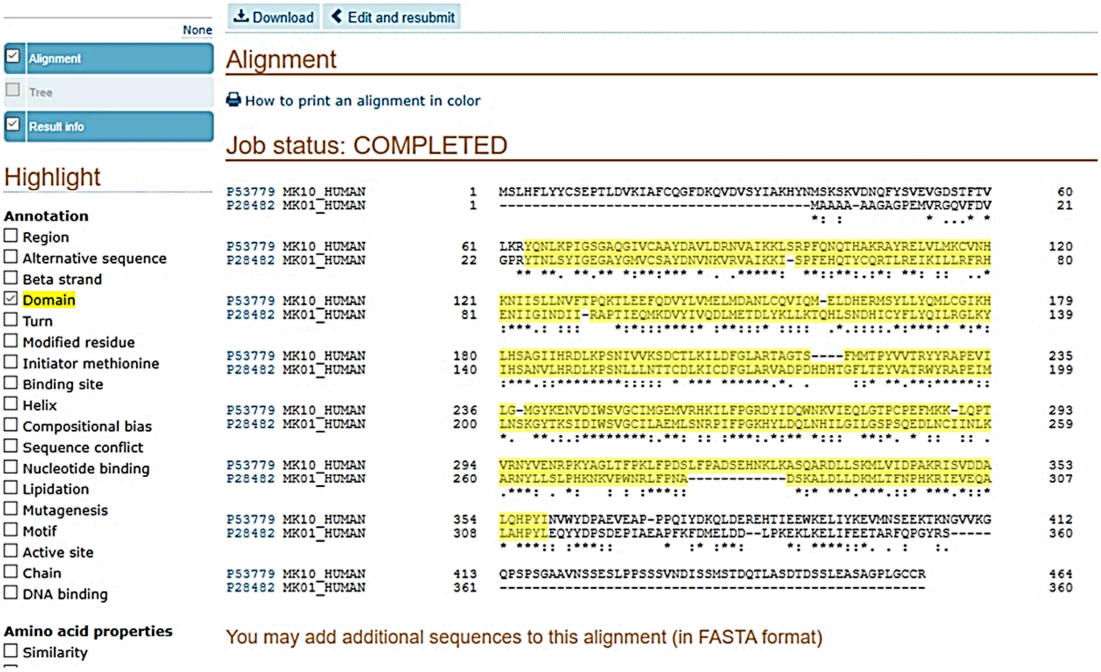

4. MAPK3 & MAPK10

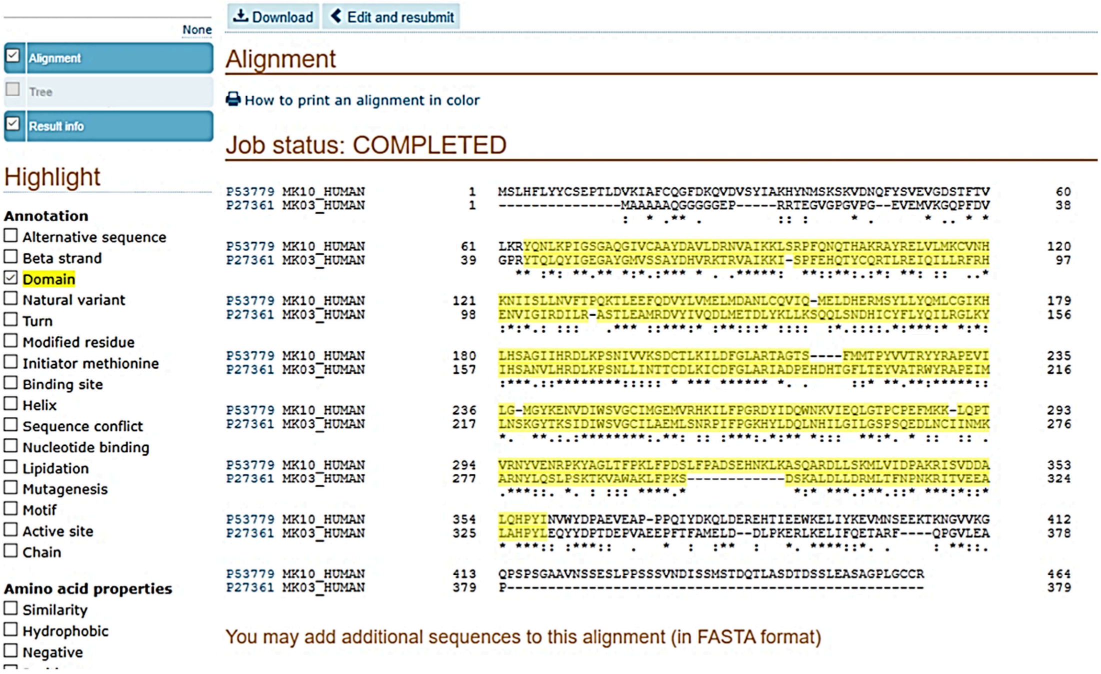

S5 - Alignment of four proteins

"Yellow" refers to JNK3 75-100 amino acid residues
"Red" refers to JNK1 1-60 amino acid residues
"Green" refers to the common conserved regions
"Labelled in box" refers to the conserved amino residues in the common interacting regions

An * (asterisk) indicates positions which have a single, fully conserved residue
A : (colon) indicates conservation between groups of strongly similar properties - scoring > 0.5 in the Gonnet PAM 250 matrix.
A . (period) indicates conservation between groups of weakly similar properties - scoring =< 0.5 in the Gonnet PAM 250 matrix.

The sequence pattern "G:V.:AYDX:X.X.VAIKK:SXP" is supposed to be the region that interacts with the N-terminal of P16INK4A

Alignment was done using the UniProt Align function (https://www.uniprot.org/align/)

CLUSTAL O(1.2.4) multiple sequence alignment

```
SP|P53779|MK10_HUMAN MSLHFLYYCSEPTLDVKIAFCQGFDKQVDVSYIAKHYNMSKSKVDNQFYSVEVGDSTFTV 60
SP|P45983|MK08_HUMAN ---------------------------------------MSRSKRDNNFYSVEIGDSTFTV 22
SP|P28482|MK01_HUMAN ---------------MAAAAA------------------------AGAGPEMVRGQVFDV 21
SP|P27361|MK03_HUMAN ---------------MAAAAAQGGGGGEPR----RT---EGVGPGVPGEVEMVKGQPFDV 38
                                                                         : .. * *

SP|P53779|MK10_HUMAN LKRYQNLKPIGSGAQGIVCAAYDAVLDRNVAIKKLSRPFQNQTHAKRAYRELVLMKCVNH 120
SP|P45983|MK08_HUMAN LKRYQNLKPIGSGAQGIVCAAYDAILERNVAIKKLSRPFQNQTHAKRAYRELVLMKCVNH 82
SP|P28482|MK01_HUMAN GPRYTNLSYIGEGAYGMVCSAYDNVNKVRVAIKKIS-PFEHQTYCQRTLREIKILLRFRH 80
SP|P27361|MK03_HUMAN GPRYTQLQYIGEGAYGMVSSAYDHVRKTRVAIKKIS-PFEHQTYCQRTLREIQILLRFRH 97
                       ** :*. **.** *:*.:*** : . .*****:* **::**:.:*: **: ::  ..*

SP|P53779|MK10_HUMAN KNIISLLNVFTPQKTLEEFQDVYLVMELMDANLCQVIQ-MELDHERMSYLLYQMLCGIKH 179
SP|P45983|MK08_HUMAN KNIIGLLNVFTPQKSLEEFQDVYIVMELMDANLCQVIQ-MELDHERMSYLLYQMLCGIKH 141
SP|P28482|MK01_HUMAN ENIIGINDIIR-APTIEQMKDVYIVQDLMETDLYKLLKTQHLSNDHICYFLYQILRGLKY 139
SP|P27361|MK03_HUMAN ENVIGIRDILR-ASTLEAMRDVYIVQDLMETDLYKLLKSQQLSNDHICYFLYQILRGLKY 156
                     :*:*.: :::    ::* ::***:* :**:::* ::::  .*.::::.*:***:* *:*:

SP|P53779|MK10_HUMAN LHSAGIIHRDLKPSNIVVKSDCTLKILDFGLARTAGTS----FMMTPYVVTRYYRAPEVI 235
```

```
SP|P45983|MK08_HUMAN LHSAGIIHRDLKPSNIVVKSDCTLKILDFGLARTAGTS----FMMTPYVVTRYYRAPEVI 197
SP|P28482|MK01_HUMAN IHSANVLHRDLKPSNLLLNTTCDLKICDFGLARVADPDHDHTGFLTEYVATRWYRAPEIM 199
SP|P27361|MK03_HUMAN IHSANVLHRDLKPSNLLINTTCDLKICDFGLARIADPEHDHTGFLTEYVATRWYRAPEIM 216
                     :***.::********::::: * *** ****** *. .     ::* **.**:*****::

SP|P53779|MK10_HUMAN LG-MGYKENVDIWSVGCIMGEMVRHKILFPGRDYIDQWNKVIEQLGTPCPEFMKK-LQPT 293
SP|P45983|MK08_HUMAN LG-MGYKENVDLWSVGCIMGEMVCHKILFPGRDYIDQWNKVIEQLGTPCPEFMKK-LQPT 255
SP|P28482|MK01_HUMAN LNSKGYTKSIDIWSVGCILAEMLSNRPIFPGKHYLDQLNHILGILGSPSQEDLNCIINLK 259
SP|P27361|MK03_HUMAN LNSKGYTKSIDIWSVGCILAEMLSNRPIFPGKHYLDQLNHILGILGSPSQEDLNCIINMK 276
                     *.  **.:.:*:******:.**: :: :***:.*:** *:::  **:*. * ::  :: .

SP|P53779|MK10_HUMAN VRNYVENRPKYAGLTFPKLFPDSLFPADSEHNKLKASQARDLLSKMLVIDPAKRISVDDA 353
SP|P45983|MK08_HUMAN VRTYVENRPKYAGYSFEKLFPDVLFPADSEHNKLKASQARDLLSKMLVIDASKRISVDEA 315
SP|P28482|MK01_HUMAN ARNYLLSLPHKNKVPWNRLFPNA------------DSKALDLLDKMLTFNPHKRIEVEQA 307
SP|P27361|MK03_HUMAN ARNYLQSLPSKTKVAWAKLFPKS------------DSKALDLLDRMLTFNPNKRITVEEA 324
                     .*.*: . *      : :***.              *:* ***.:**.::  *** *::*

SP|P53779|MK10_HUMAN LQHPYINVWYDPAEVEAP-PPQIYDKQLDEREHTIEEWKELIYKEVMNSEEKTKNGVVKG 412
SP|P45983|MK08_HUMAN LQHPYINVWYDPSEAEAP-PPKIPDKQLDEREHTIEEWKELIYKEVMDLEERTKNGVIRG 374
SP|P28482|MK01_HUMAN LAHPYLEQYYDPSDEPIAEAPFKFDMELD--DLPKEKLKELIFEETARFQP----GYRS- 360
SP|P27361|MK03_HUMAN LAHPYLEQYYDPTDEPVAEEPFTFAMELD--DLPKERLKELIFQETARFQP----GVLEA 378
                     * ***:: :***::      *     :**  :   *. ****::*.   :     *

SP|P53779|MK10_HUMAN QPSPSGAAVNSSESL-PPSSSVNDISSMSTDQTLASDTDSSLEASAGPLGCCR 464
SP|P45983|MK08_HUMAN QPSPLGAAVINGSQHPSSSSSVNDVSSMSTDPTLASDTDSSLEAAAGPLGCCR 427
SP|P28482|MK01_HUMAN -----------------------------------------------------
SP|P27361|MK03_HUMAN P---------------------------------------------------- 379
```

Amino acid sequence similarity through the 4 proteins

```
P53779 MK10_HUMAN      1   MSLHFLYYCSEPTLDVKIAFCQGFDKQVDVSYIAKHYNMSKSKVDNQFYSVEVGDSTFTV    60
P45983 MK08_HUMAN      1   --------------------------------------MSRSKRDNNFYSVEIGDSTFTV    22
P28482 MK01_HUMAN      1   ---------------MAAAAA------------------------AGAGPEMVRGQVFDV    21
P27361 MK03_HUMAN      1   ---------------MAAAAAQGGGGGEPR----RT---EGVGPGVPGEVEMVKGQPFDV    38
                                                                               : .. * *

P53779 MK10_HUMAN     61   LKRYQNLKPIGSGAQGIVCAAYDAVLDRNVAIKKLSRPFQNQTHAKRAYRELVLMKCVNH   120
P45983 MK08_HUMAN     23   LKRYQNLKPIGSGAQGIVCAAYDAILERNVAIKKLSRPFQNQTHAKRAYRELVLMKCVNH    82
P28482 MK01_HUMAN     22   GPRYTNLSYIGEGAYGMVCSAYDNVNKVRVAIKKIS-PFEHQTYCQRTLREIKILLRFRH    80
P27361 MK03_HUMAN     39   GPRYTQLQYIGEGAYGMVSSAYDHVRKTRVAIKKIS-PFEHQTYCQRTLREIQILLRFRH    97
                              ** :*. **.** *:*.:*** : . .*****:* **::**:.:*: **: ::  ..*

P53779 MK10_HUMAN    121   KNIISLLNVFTPQKTLEEFQDVYLVMELMDANLCQVIQ-MELDHERMSYLLYQMLCGIKH   179
P45983 MK08_HUMAN     83   KNIIGLLNVFTPQKSLEEFQDVYIVMELMDANLCQVIQ-MELDHERMSYLLYQMLCGIKH   141
P28482 MK01_HUMAN     81   ENIIGINDIIR-APTIEQMKDVYIVQDLMETDLYKLLKTQHLSNDHICYFLYQILRGLKY   139
P27361 MK03_HUMAN     98   ENVIGIRDILR-ASTLEAMRDVYIVQDLMETDLYKLLKSQQLSNDHICYFLYQILRGLKY   156
                            :*:*.: :::    ::* ::***:* :**:::* ::::  .*.::::.*:***:* *:*:

P53779 MK10_HUMAN    180   LHSAGIIHRDLKPSNIVVKSDCTLKILDFGLARTAGTS----FMMTPYVVTRYYRAPEVI   235
P45983 MK08_HUMAN    142   LHSAGIIHRDLKPSNIVVKSDCTLKILDFGLARTAGTS----FMMTPYVVTRYYRAPEVI   197
P28482 MK01_HUMAN    140   IHSANVLHRDLKPSNLLLNTTCDLKICDFGLARVADPDHDHTGFLTEYVATRWYRAPEIM   199
P27361 MK03_HUMAN    157   IHSANVLHRDLKPSNLLINTTCDLKICDFGLARIADPEHDHTGFLTEYVATRWYRAPEIM   216
                            :***.::********::::: * *** ****** *. .     ::* **.**:*****::

P53779 MK10_HUMAN    236   LG-MGYKENVDIWSVGCIMGEMVRHKILFPGRDYIDQWNKVIEQLGTPCPEFMKK-LQPT   293
P45983 MK08_HUMAN    198   LG-MGYKENVDLWSVGCIMGEMVCHKILFPGRDYIDQWNKVIEQLGTPCPEFMKK-LQPT   255
P28482 MK01_HUMAN    200   LNSKGYTKSIDIWSVGCILAEMLSNRPIFPGKHYLDQLNHILGILGSPSQEDLNCIINLK   259
P27361 MK03_HUMAN    217   LNSKGYTKSIDIWSVGCILAEMLSNRPIFPGKHYLDQLNHILGILGSPSQEDLNCIINMK   276
                            *.  **.:.:*:******:.**: :: :***:.*:** *:::  **:*. * ::  :: .

P53779 MK10_HUMAN    294   VRNYVENRPKYAGLTFPKLFPDSLFPADSEHNKLKASQARDLLSKMLVIDPAKRISVDDA   353
P45983 MK08_HUMAN    256   VRTYVENRPKYAGYSFEKLFPDVLFPADSEHNKLKASQARDLLSKMLVIDASKRISVDEA   315
P28482 MK01_HUMAN    260   ARNYLLSLPHKNKVPWNRLFPNA------------DSKALDLLDKMLTFNPHKRIEVEQA   307
P27361 MK03_HUMAN    277   ARNYLQSLPSKTKVAWAKLFPKS------------DSKALDLLDRMLTFNPNKRITVEEA   324
                            .*.*: . *      : :***.              *:* ***.:**.::  *** *::*

P53779 MK10_HUMAN    354   LQHPYINVWYDPAEVEAP-PPQIYDKQLDEREHTIEEWKELIYKEVMNSEEKTKNGVVKG   412
P45983 MK08_HUMAN    316   LQHPYINVWYDPSEAEAP-PPKIPDKQLDEREHTIEEWKELIYKEVMDLEERTKNGVIRG   374
P28482 MK01_HUMAN    308   LAHPYLEQYYDPSDEPIAEAPFKFDMELD--DLPKEKLKELIFEETARFQP----GYRS-   360
P27361 MK03_HUMAN    325   LAHPYLEQYYDPTDEPVAEEPFTFAMELD--DLPKERLKELIFQETARFQP----GVLEA   378
                            * ***:: :***::      *     :**  :   *. ****::*.    :     *

P53779 MK10_HUMAN    413   QPSPSGAAVNSSESL-PPSSSVNDISSMSTDQTLASDTDSSLEASAGPLGCCR        464
P45983 MK08_HUMAN    375   QPSPLGAAVINGSQHPSSSSSVNDVSSMSTDPTLASDTDSSLEAAAGPLGCCR        427
P28482 MK01_HUMAN    361   -----------------------------------------------------        360
P27361 MK03_HUMAN    379   P----------------------------------------------------        379
```

Predicted conserved domain through the 4 proteins

```
P53779 MK10_HUMAN      1   MSLHFLYYCSEPTLDVKIAFCQGFDKQVDVSYIAKHYNMSKSKVDNQFYSVEVGDSTFTV   60
P45983 MK08_HUMAN      1   --------------------------------------MSRSKRDNNFYSVEIGDSTFTV   22
P28482 MK01_HUMAN      1   ---------------MAAAAA-----------------------AGAGPEMVRGQVFDV   21
P27361 MK03_HUMAN      1   ---------------MAAAAAQGGGGGEPR----RT---EGVGPGVPGEVEMVKGQPFDV   38
                                                                        : ..  * *

P53779 MK10_HUMAN     61   LKRYQNLKPIGSGAQGIVCAAYDAVLDRNVAIKKLSRPFQNQTHAKRAYRELVLMKCVNH  120
P45983 MK08_HUMAN     23   LKRYQNLKPIGSGAQGIVCAAYDAILERNVAIKKLSRPFQNQTHAKRAYRELVLMKCVNH   82
P28482 MK01_HUMAN     22   GPRYTNLSYIGEGAYGMVCSAYDNVNKVRVAIKKIS-PFEHQTYCQRTLREIKILLRFRH   80
P27361 MK03_HUMAN     39   GPRYTQLQYIGEGAYGMVSSAYDHVRKTRVAIKKIS-PFEHQTYCQRTLREIQILLRFRH   97
                              ** :*. **.** *:*.:*** : . .*****:* **::**:.:*: **: ::  ..*

P53779 MK10_HUMAN    121   KNIISLLNVFTPQKTLEEFQDVYLVMELMDANLCQVIQ-MELDHERMSYLLYQMLCGIKH  179
P45983 MK08_HUMAN     83   KNIIGLLNVFTPQKSLEEFQDVYIVMELMDANLCQVIQ-MELDHERMSYLLYQMLCGIKH  141
P28482 MK01_HUMAN     81   ENIIGINDIIR-APTIEQMKDVYIVQDLMETDLYKLLKTQHLSNDHICYFLYQILRGLKY  139
P27361 MK03_HUMAN     98   ENVIGIRDILR-ASTLEAMRDVYIVQDLMETDLYKLLKSQQLSNDHICYFLYQILRGLKY  156
                          :*:*.: :::    ::* ::***:* :**:::* ::::  .*.::::.*:***:* *:*:

P53779 MK10_HUMAN    180   LHSAGIIHRDLKPSNIVVKSDCTLKILDFGLARTAGTS----FMMTPYVVTRYYRAPEVI  235
P45983 MK08_HUMAN    142   LHSAGIIHRDLKPSNIVVKSDCTLKILDFGLARTAGTS----FMMTPYVVTRYYRAPEVI  197
P28482 MK01_HUMAN    140   IHSANVLHRDLKPSNLLLNTTCDLKICDFGLARVADPDHDHTGFLTEYVATRWYRAPEIM  199
P27361 MK03_HUMAN    157   IHSANVLHRDLKPSNLLINTTCDLKICDFGLARIADPEHDHTGFLTEYVATRWYRAPEIM  216
                          :***.::********:::: * *** ****** *. .     ::* **.**:*****::

P53779 MK10_HUMAN    236   LG-MGYKENVDIWSVGCIMGEMVRHKILFPGRDYIDQWNKVIEQLGTPCPEFMKK-LQPT  293
P45983 MK08_HUMAN    198   LG-MGYKENVDLWSVGCIMGEMVCHKILFPGRDYIDQWNKVIEQLGTPCPEFMKK-LQPT  255
P28482 MK01_HUMAN    200   LNSKGYTKSIDIWSVGCILAEMLSNRPIFPGKHYLDQLNHILGILGSPSQEDLNCIINLK  259
P27361 MK03_HUMAN    217   LNSKGYTKSIDIWSVGCILAEMLSNRPIFPGKHYLDQLNHILGILGSPSQEDLNCIINMK  276
                          *.  **.:.:*:******:.**: :: :***:.*:** *:::  **:*. * ::  :: .

P53779 MK10_HUMAN    294   VRNYVENRPKYAGLTFPKLFPDSLFPADSEHNKLKASQARDLLSKMLVIDPAKRISVDDA  353
P45983 MK08_HUMAN    256   VRTYVENRPKYAGYSFEKLFPDVLFPADSEHNKLKASQARDLLSKMLVIDASKRISVDEA  315
P28482 MK01_HUMAN    260   ARNYLLSLPHKNKVPWNRLFPNA------------DSKALDLLDKMLTFNPHKRIEVEQA  307
P27361 MK03_HUMAN    277   ARNYLQSLPSKTKVAWAKLFPKS------------DSKALDLLDRMLTFNPNKRITVEEA  324
                          .*.*: . *      : :***.              *:* ***.:**.::  *** *::*

P53779 MK10_HUMAN    354   LQHPYINVWYDPAEVEAP-PPQIYDKQLDEREHTIEEWKELIYKEVMNSEEKTKNGVVKG  412
P45983 MK08_HUMAN    316   LQHPYINVWYDPSEAEAP-PPKIPDKQLDEREHTIEEWKELIYKEVMDLEERTKNGVIRG  374
P28482 MK01_HUMAN    308   LAHPYLEQYYDPSDEPIAEAPFKFDMELD--DLPKEKLKELIFEETARFQP----GYRS-  360
P27361 MK03_HUMAN    325   LAHPYLEQYYDPTDEPVAEEPFTFAMELD--DLPKERLKELIFQETARFQP----GVLEA  378
                          * ***:: :***::      *     :**  :   *. ****::*.   :     *

P53779 MK10_HUMAN    413   QPSPSGAAVNSSESL-PPSSSVNDISSMSTDQTLASDTDSSLEASAGPLGCCR        464
P45983 MK08_HUMAN    375   QPSPLGAAVINGSQHPSSSSSVNDVSSMSTDPTLASDTDSSLEAAAGPLGCCR        427
P28482 MK01_HUMAN    361   -----------------------------------------------------        360
P27361 MK03_HUMAN    379   P----------------------------------------------------        379
```
